# Decarboxylation via a Higher Electronic Excited State Drives LSSmOrange Photoconversion

**DOI:** 10.1021/acsphyschemau.6c00009

**Published:** 2026-04-12

**Authors:** Hyang Sook Seol, Fangjia Luo, Elke De Zitter, Nipawan Nuemket, Eduard Fron, Neil R. McFarlane, Leonie De Vrieze, Janko Civic, Michiel Postelmans, Savannah Van Bel, Shigeki Owada, Kensuke Tono, Tomoyuki Tanaka, Toshi Arima, Hiroki Noguchi, Thi Yen Hang Bui, Rie Tanaka, Kazuya Hasegawa, Kunio Hirata, Dohyun Im, Tsuyoshi Araya, Tetsunari Kimura, Luc Van Meervelt, Martin Weik, Jeremy N. Harvey, Jacques-Philippe Colletier, So Iwata, Eriko Nango, Hideaki Mizuno

**Affiliations:** † Laboratory of Biomolecular Network Dynamics, Biochemistry, Molecular and Structural Biology Section, Department of Chemistry, 26657 KU Leuven, Celestijnenlaan 200G bus 2403, Leuven 3001, Belgium; ‡ 133704 Japan Synchrotron Radiation Research Institute, 1-1-1 Kouto, Sayo, Hyogo 679-5198, Japan; § Univ. Grenoble Alpes, CEA, CNRS, 55543 Institut de Biologie Structurale, Grenoble 38000, France; ∥ SPring-8 Center, 13593 RIKEN, 1-1-1 Kouto, Sayo-cho, Sayo-gun, Hyogo 679-5148, Japan; ⊥ Chem & Tech-Molecular Imaging and Photonics, Department of Chemistry, KU Leuven, Celestijnenlaan 200F bus 2404, Leuven 3001, Belgium; # Quantum Chemistry and Physical Chemistry Section, Department of Chemistry, KU Leuven, Celestijnenlaan 200F bus 2404, Leuven 3001, Belgium; ¶ Laboratory of Biomolecular Modelling and Design, Biochemistry, Molecular and Structural Biology Section, Department of Chemistry, KU Leuven, Celestijnenlaan 200G bus 2403, Leuven 3001, Belgium; ∇ Laboratory of Biomolecular Architecture, Biochemistry, Molecular and Structural Biology Section, Department of Chemistry, KU Leuven, Celestijnenlaan 200G bus 2403, Leuven 3001, Belgium; ○ Department of Cell Biology, Graduate School of Medicine, Kyoto University, Yoshidakonoe-cho, Sakyo-ku, Kyoto 606-8501, Japan; ⧫ Department of Chemistry, Graduate School of Science, Kobe University, 1-1 Rokkodai, Nada, Kobe 657-8501, Japan; †† Institute of Multidisciplinary Research for Advanced Materials, 13101 Tohoku University, Aoba-ku, Sendai 980-8577, Japan

**Keywords:** photoswitchable fluorescent proteins, Kolbe decarboxylation, time-resolved serial femtosecond crystallography, time-resolved spectroscopy, X-ray free electron laser

## Abstract

LSSmOrange is a fluorescent protein that exhibits slow photoconversion and is used as an imaging tool to highlight specific subpopulations of molecules. While photoconversion in LSSmOrange is known to involve Kolbe decarboxylation of the E215 side chain, its structural dynamics remain unexplored. We addressed the excited state dynamics of LSSmOrange by using serial femtosecond crystallography (SFX) with an X-ray free electron laser. SFX enabled us to determine the crystal structure of unconverted LSSmOrange without detectable X-ray damage, facilitating a femtosecond optical pump—X-ray probe experiment to track time-resolved structural changes. A decrease in electron density at E215 was observed 250 ps after strong pump laser illumination (mean fluence of 0.31 J/cm^2^), consistent with photoconversion by decarboxylation. Extrapolated structures suggested the appearance of an alternative E215 conformation in addition to illumination-induced decarboxylation. Since the photoinduced decarboxylation occurred despite the extremely low single-photon photoconversion quantum yield of LSSmOrange (8.5 × 10^–6^), we hypothesized that the photoconversion proceeds via a multiphoton process. Power titration using transient absorption spectroscopy revealed that photoconversion was linked to optical nonlinearity under a high pump laser energy. We propose that LSSmOrange photoconversion involves a higher-lying electronic excited state triggered by multiphoton absorption, as supported by quantum-chemical calculations. Photoconversion via a multiphoton process enables spatially confined highlighting on a microscope.

## Graphical abstract

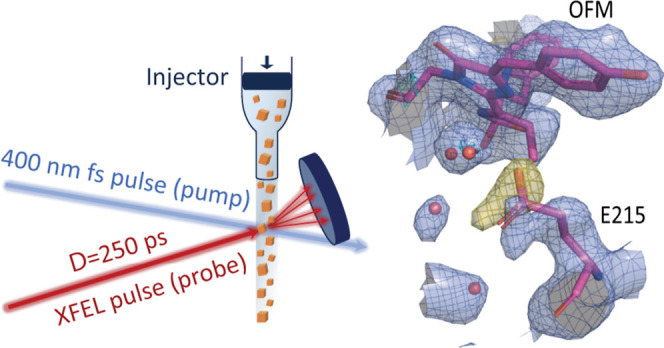

## Introduction

Fluorescent proteins are genetically encoded markers applicable for noninvasive protein labeling. They are widely used for imaging in living specimens and have become indispensable tools in the field of life sciences. Certain fluorescent proteins show a large spectrum shift (LSS) between their absorption and emission bands (>100 nm).^1–3^ These fluorescent proteins enable dual fluorescence detection with a single excitation wavelength when used in combination with a fluorescent protein exhibiting a standard Stokes shift.^1,3^ LSSmOrange was engineered from mOrange to incorporate the LSS property and has absorption and emission maxima at 437 and 572 nm, respectively.^3^ This big energy gap originates from excited-state proton transfer (ESPT).^4^ The chromophore of LSSmOrange is predominantly in a neutral form under physiological pH conditions. Upon excitation, a proton is abstracted from the chromophore via ESPT, and the resulting anionic chromophore emits an orange fluorescence. Additionally, LSSmOrange can undergo photoconversion with an irreversible absorption band shift from 437 to 553 nm that has been assigned to the formation of the deprotonated anionic chromophore in the ground state.^4,5^ LSSmOrange photoconversion is characterized by an extremely low quantum yield (8.5 × 10^–6^), explaining why photoconversion is elicited only upon intense 400 nm illumination.^4,5^ The photoconversion is proposed to occur via a Kolbe-type mechanism, involving the photoinduced electron transfer from the E215 carboxylate to the chromophore (numbering adopted from the PDB file of LSSmOrange, 4Q7R), followed by radical-mediated decarboxylation and subsequent back electron transfer.^4^ This property of LSSmOrange is useful in applications such as the analysis of molecular diffusion and optical highlighting of subcellular organelles in living cells.^5^

A crystal structure of LSSmOrange has been solved at a cryogenic temperature (100 K) with crystals grown at pH 4.5 (PDB: 4Q7R).^6^ The structure revealed that the 4-(*p*-hydroxybenzylidene)-5-imidazolinone chromophore originates from the T66-Y67-G68 tripeptide and adopts the *cis* configuration, as commonly observed in various fluorescent proteins (Figure 1). In addition, the π-conjugation system of the chromophore extends to the oxazoline ring formed by the cyclization of the T66 side chain. The hydroxyl moiety of the chromophore forms a hydrogen bond with the side chain of S146, which in turn forms a hydrogen bond with the carboxylate group of D161. This network has been proposed as an ESPT pathway.

**Figure 1:**
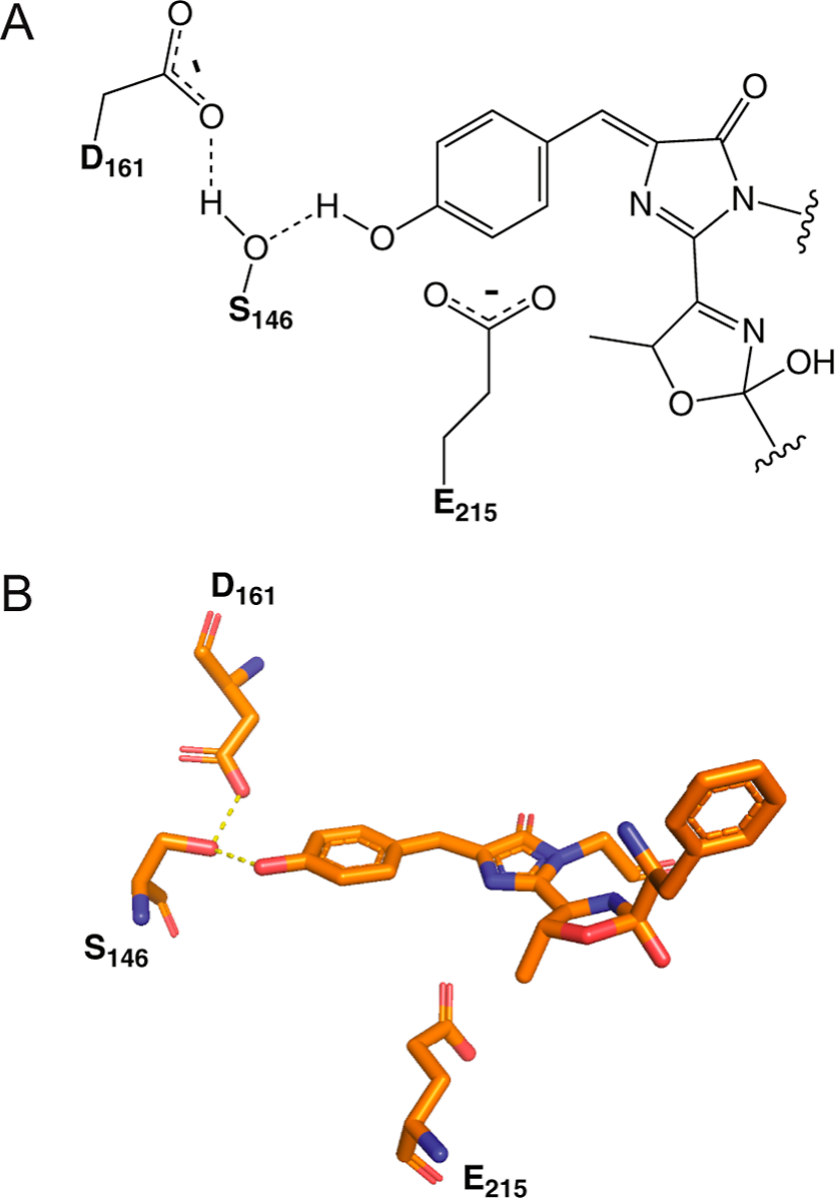
Chromophore structure of LSSmOrange. (A) The chromophore, 2-[(4-)-5-methyl-2-hydroxy-3-oxazoline]-4-(*p*-hydroxybenzylidene)-5-imidazolinone, is composed of the tripeptide T66-Y67-G68. Three critical side chains around the chromophore are also shown (S146, D161, E215). (B) Reported cryogenic structure of the chromophore and three key side chains at pH 4.5 (PDB: 4Q7R).^6^ Dashed lines indicate hydrogen bonds.

Although this structural information provides important insights, the fact that the structure was solved at 100 K using crystals grown at pH 4.5 raises questions about the relevance of the observed chromophore state and coordination in the context of fluorescence emission and photoconversion mechanisms.^6^ Indeed, in this study, we observed a dim fluorescence of LSSmOrange under acidic conditions and a significant spectral change under cryogenic conditions. Therefore, an LSSmOrange structure solved at room temperature (RT) under neutral pH conditions is necessary to examine the chromophore coordination in environments similar to those used in imaging applications.

In this study, we determined the crystal structure of unconverted LSSmOrange at neutral pH and by RT by serial femtosecond crystallography (SFX) with an X-ray free electron laser (XFEL). An XFEL provides coherent femtosecond X-ray pulses with high peak brilliance, enabling the recording of a single diffraction pattern prior to the onset of radiation damage in the crystal at RT.^7–9^ By serially collecting diffraction patterns from tens of thousands of crystals, SFX allows for the determination of crystal structures while mitigating X-ray radiation damage.^10^ By combining SFX with a femtosecond pulse laser as an optical pump (time-resolved SFX; TR-SFX),^11^ we investigated the photoinduced changes in electron density of LSSmOrange, which are presumed to be responsible for photoconversion. We then spectroscopically evaluated the contribution of optical nonlinearity to the efficient photoconversion of LSSmOrange upon illumination with a 400 nm femtosecond laser.

## Results and Discussion

### Additional LSSmOrange Protonation under Acidic Conditions

As reported previously,^4^ the chromophore of LSSmOrange is predominantly protonated at neutral pH and the absorption band corresponding to the deprotonated chromophore appears only at alkaline pH. Thus, the p*K*_a_ of the chromophore itself is expected to be in the alkaline range (>11). However, Shcherbakova et al. reported an inconsistent p*K*_a_ value in the acidic range (5.7) by performing a titration based on fluorescence, rather than absorption.^3^ This led to the assumption that an additional protonation of the protein could occur under acidic conditions, resulting in diminished fluorescence. To understand the protonation states, we acquired the absorption spectra of LSSmOrange at varying pH values in solution at RT (Figure 2A). In the neutral pH range, a single absorption band at 438 nm, corresponding to the protonated neutral chromophore, was observed in addition to the common protein absorption band at around 280 nm.^4^ At pH 10.0 or higher, a small absorption band corresponding to the deprotonated anionic chromophore appeared at 554 nm while the absorption of the neutral chromophore remained dominant (Figure 2A,B).^4^ Hence, the p*K*_a_ associated with chromophore deprotonation (p*K*_a2_) appeared to be higher than 11 (Figure 2B), but the exact value could not be determined due to protein denaturation occurring above pH 11.0. Below pH 5.0, the main absorption peak exhibited a slight blue-shift from 438 to 426 nm (Figure 2C). By performing a pH titration over the wavelength shift, the apparent p*K*_a_ value of the additional protonation was determined to be 4.25 ± 0.13. Based on these observations, we concluded that the chromophore is in a protonated neutral state under neutral pH conditions, while an additional protonation of the protein under acidic conditions induces the blue-shift of the absorption band.

**Figure 2:**
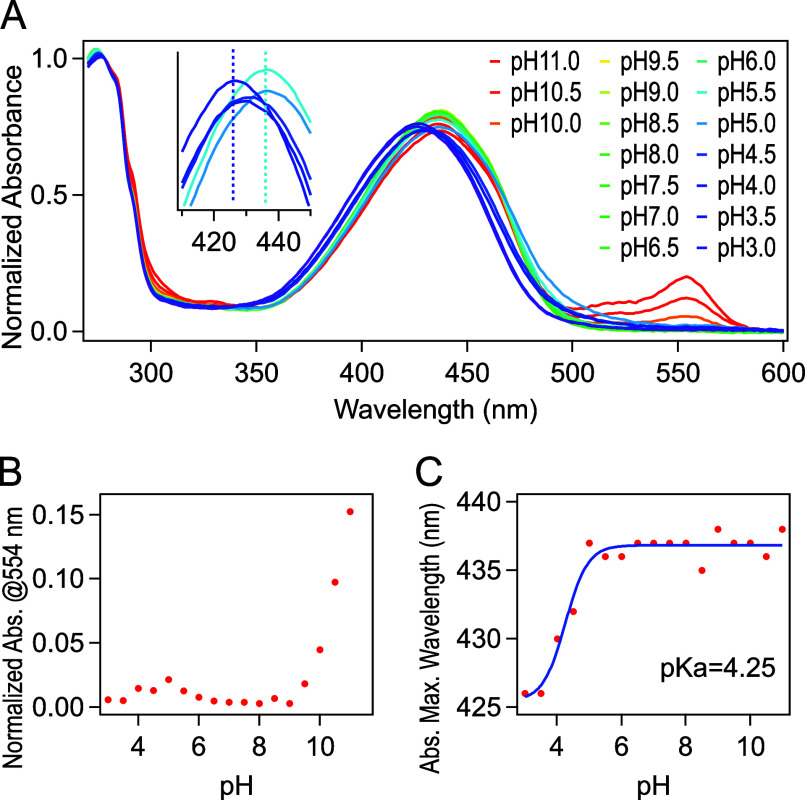
Absorption spectra of LSSmOrange under various pH conditions. (A) Overlaid absorption spectra normalized at 278 nm. The spectra were acquired in 50 mM citrate (pH 3.0–6.5), HEPES (pH 7.0–8.5), or glycine buffers (pH 9.0–11.0) containing 150 mM NaCl. The concentration of LSSmOrange was 0.13 mM. The inset shows the expansion of the peaks around 420–440 nm (pH 3.5–5.5). (B) Appearance of the peak at 554 nm corresponding to the deprotonated anionic chromophore. p*K*_a2_ was not determinable since the absorbance did not reach the plateau level at pH 11.0. (C) pH-dependent shift of the main peak around 420–440 nm. A sigmoidal fit is shown in blue. The apparent p*K*_a1_ was calculated to be 4.25 ± 0.13. Note that the apparent p*K*_a1_ might be deviating from the intrinsic value since the linear correlation could not be guaranteed between the maximum wavelength and the concentration of protonated or deprotonated species.

### Fluorescence Properties of Doubly Protonated LSSmOrange

We next examined the fluorescence properties of LSSmOrange in solution by collecting fluorescence spectra at various pH. Upon the excitation of the main absorption band at 440 nm at pH 8.0, an emission peak appeared at 573 nm with a shoulder around 610 nm (Figure 3A). This band was assigned to the emission from the excited-state anionic chromophore.^4^ A single excitation peak for this emission was observed at 437 nm, which corresponded to the absorption band of the neutral-state chromophore. Although the absorbance under acidic conditions was comparable to that under neutral conditions, the fluorescence was significantly reduced as the pH decreased (Figure 3B). For example, the fluorescence intensity at pH 3.0 was only 4.1% of that at pH 8.0 (excitation at 438 nm, emission at 570 nm). In contrast to the absorption spectra, a pH-dependent blue-shift of the excitation peak was not observed. The intrinsic p*K*_a1_ was determined to 5.11 ± 0.07 (Figure 3C) by a pH titration based on fluorescence intensity (excitation at 438 nm, emission at 570 nm), which was in good agreement with the p*K*_a_ value reported by Shcherbakova et al. (5.7).^3^

**Figure 3:**
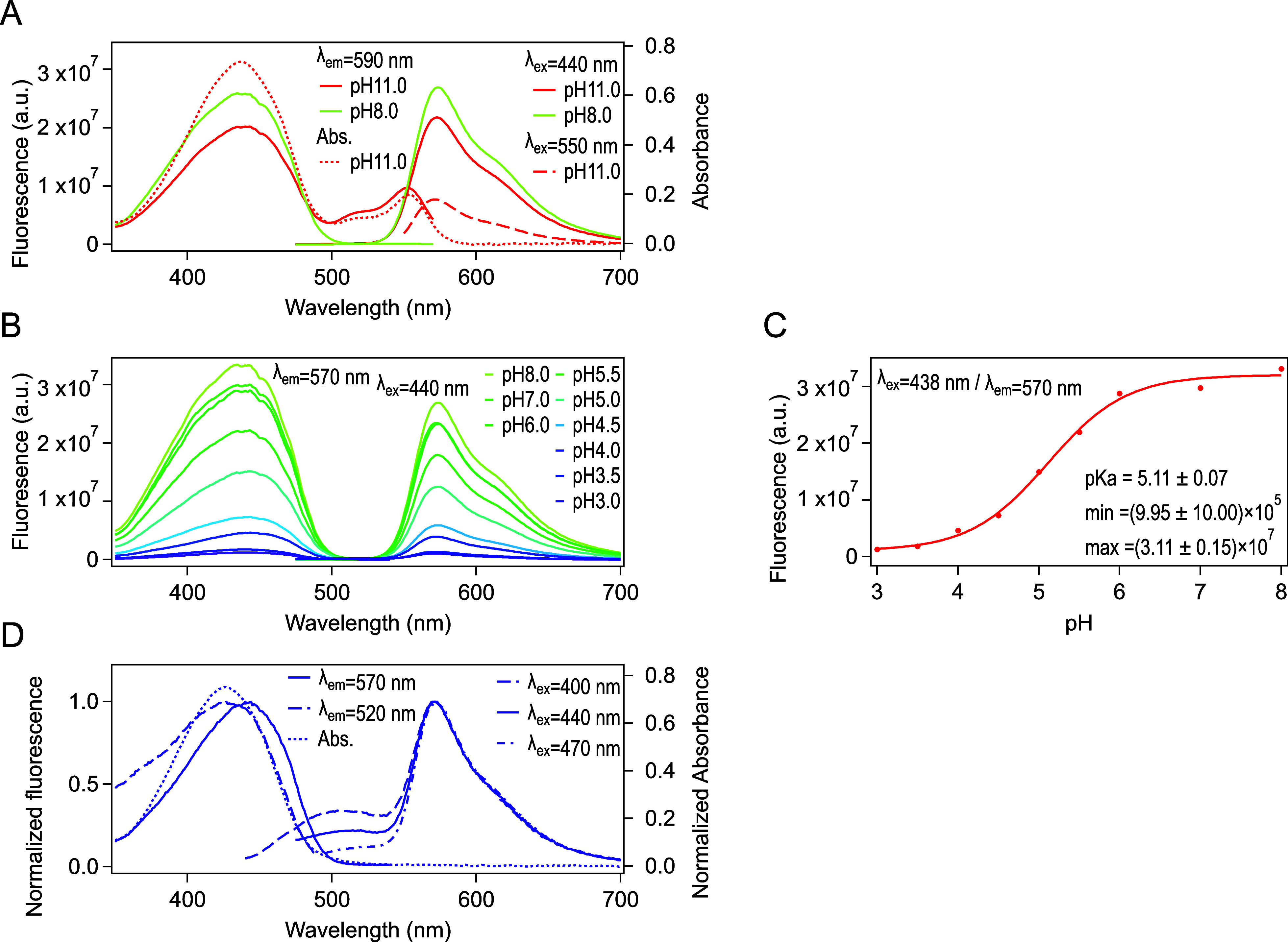
Fluorescence spectra of LSSmOrange at RT. (A) Excitation (left) and emission spectra (right) at pH 8.0 (greenish yellow) and pH 11.0 (red). The excitation spectra were recorded at 590 nm, whereas the emission spectra were recorded upon excitation at 440 nm (solid line) and 550 nm (dashed line). The absorption spectrum at pH 11.0 (dotted line) is overlaid. (B) pH dependency of the excitation and emission spectra from acidic to neutral pH. (C) pH titration curve of the state that showed ESPT. Fluorescence intensity emitted from the anionic state (570 nm) upon excitation of the neutral form (at 438 nm) was used for the titration. A sigmoidal fit is overlaid. The obtained (intrinsic) p*K*_a1_ was 5.11. (D) Peak-normalized excitation (left) and emission (right) spectra at pH 3.0. The excitation spectra were recorded at a detection wavelength of 570 nm (solid line) and 520 nm (dashed line). The absorption spectrum at pH 3.0 (dotted line) is overlaid. The emission spectra were recorded using excitation at 400 nm (dashed line), 440 nm (solid line), and 470 nm (dash-dotted line). The concentration of LSSmOrange was 41 μM for all measurements.

Orange fluorescence at 573 nm was dim yet still observable at pH 3.0 upon 440 nm excitation (Figure 3B). In addition, a weak green emission band appeared at 504 nm (Figure 3D). This band became more obvious with shorter excitation wavelengths, while orange fluorescence at 573 nm remained dominant. The excitation band of the green emission component (detected at 520 nm) exhibited a blue-shift to 427 nm compared to the one detected at 570 nm, which was consistent with the absorption maximum at pH 3.0 (Figure 3D). When the sample was cooled to 78 K, fluorescence emission at 504 nm was strongly enhanced and became predominant. This observation was likely caused by a reduction of internal conversion under the cooling conditions in the state absorbing at 426 nm and fluorescing at 504 nm (Figure 4).

**Figure 4:**
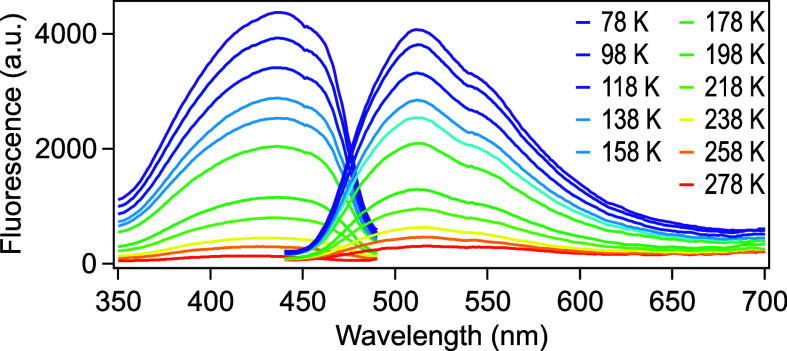
Fluorescence spectra of LSSmOrange at pH 3.0 under cryogenic conditions. LSSmOrange in solution (64 μM) in 50 mM citrate buffer (pH 3.0) was used for the measurement. The spectra were recorded at 20 K intervals from 78 K to 278 K. For the excitation spectra (left), the emission wavelength was set to 520 nm. The emission spectra (right) were recorded upon 400 nm excitation.

Overall, the fluorescence properties of LSSmOrange were governed by two protonation equilibria (p*K*_a1_ = 5.11; p*K*_a2_ >11) (Figure 5). In the physiological pH range, the singly protonated state predominated, absorbing at 437 nm and emitting bright orange fluorescence at 573 nm via ESPT.^4^ Under acidic conditions, LSSmOrange became doubly protonated, shifting its absorption peak to 426 nm and showing a faint green fluorescence at 504 nm. The weak fluorescence of this species is most likely caused by internal conversion at RT. The green range emission of this species indicated that ESPT did not occur in the excited state. These observations highlighted the significant impact of the additional protonation on the hydrogen bond network that functions as the ESPT acceptor (Figure 5). This network is composed of the chromophore hydroxyphenyl moiety, the hydroxyl group of S146, and the carboxylate group of D161. A potential second protonation site is one of the two carboxylate oxygen atoms of D161 (Figure 5B). The D161 carboxylate oxygen that is not involved in the hydrogen bond network is facing the outside of the β-barrel (Figure 7E, vide infra). The protonation of the D161 carboxylate group interferes with the ability of the group to act as the acceptor of the proton released from the chromophore due to ESPT.

**Figure 5:**
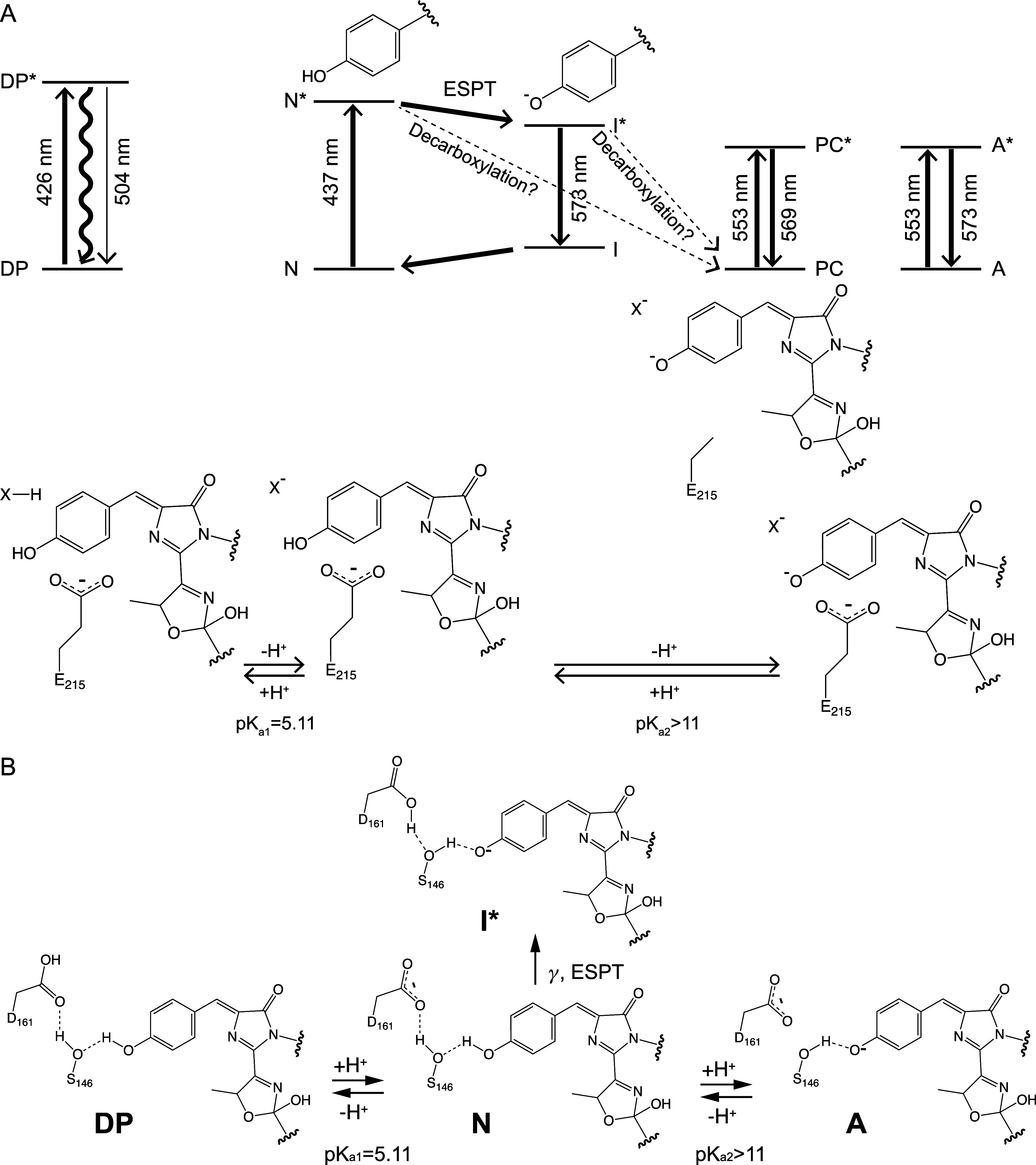
LSSmOrange protonation. (A) Schematic energy diagram of the various states of LSSmOrange. The structure of the chromophore and its surroundings are shown below the energy diagram. Asterisks indicate electronic excited states, whereas symbols without an asterisk represent ground states. The doubly protonated state (DP) displays faint fluorescence at 504 nm upon 426 nm excitation. The neutral state (N) is excited by absorbing at 437 nm, and N* undergoes ESPT resulting in the formation of the excited intermediate state (I*) that relaxes to the ground state emitting 573 nm fluorescence. The Kolbe-type decarboxylation of E215 responsible for the formation of the photoconverted state (PC) takes place upon excitation of the N state. The formed PC absorbs at 553 nm and emits at 569 nm. Under high pH conditions, the chromophore is deprotonated and is thus in the anionic state (A). This state emits 573 nm fluorescence upon 553 nm excitation. The absorption and emission wavelengths of the photoconverted state were taken from ref 4. (B) Proposed protonation states of unconverted LSSmOrange. The oxygen atom of the chromophore hydroxyphenyl moiety is part of a hydrogen bond network involving the hydroxyl group of S146 and the carboxylate group of D161. One of the carboxylate oxygens of D161 is involved in the network, and the other oxygen is facing to the outside of the β-barrel. Under neutral conditions, the chromophore is in the protonated neutral form (N). Upon excitation, the chromophore is deprotonated by ESPT and forms an anionic excited state intermediate (I*). The hydroxyl group of S146 acts as a proton relay, transferring the proton from the chromophore to the carboxylate group of D161, which serves as the proton acceptor. At low pH, D161 adopts a protonated carboxyl form, losing its ability to function as the ESPT proton acceptor. At this pH, LSSmOrange is present in the doubly protonated form (DP). At higher pH, chromophore is partially deprotonated and becomes anionic (A).

### Fluorescence Properties of LSSmOrange under Neutral and Alkaline Conditions

We investigated the fluorescence properties of LSSmOrange under neutral and alkaline conditions based on its emission and excitation spectra. At pH 11.0, an excitation peak at 553 nm with a shoulder around 520 nm appeared in the excitation spectrum recorded at 590 nm in addition to the main excitation band at 440 nm, which is characteristic of the neutral chromophore (Figure 3A). The position and shape of this additional peak were similar to the absorption peak of the anionic form chromophore at pH 11.0 (Figure 2A). The intensity of the excitation peak at 553 nm reached to 48% of that at 437 nm, while the absorbance at 553 nm was only 27% of the absorbance at 437 nm. This higher peak ratio in the excitation spectrum suggested that the chromophore in the anionic state had a higher fluorescence quantum yield than that in the neutral state.

The emission spectrum obtained by exciting the anionic chromophore (at 550 nm, pH 11.0) peaked at 573 nm, with a shoulder around 610 nm (Figure 3A), mirroring the absorption peak of the anionic chromophore at 553 nm. This indicates that the orange fluorescence at 573 nm was the result of direct emission from the anionic chromophore. The emission spectrum of the anionic form was almost identical to that of the neutral form excited at 440 nm at both pH 8.0 and 11.0. This observation supported the notion that ESPT precedes fluorescence emission after the excitation of the neutral chromophore. We concluded that a small fraction of the chromophore population was in the deprotonated anionic state under alkaline conditions, whereas the protonated chromophore was predominant in the neutral pH range. The neutral chromophore showed efficient ESPT upon excitation and relaxed to the ground state, emitting orange fluorescence from the anionic form chromophore that served as an intermediate in the excited state (Figure 5).

We also measured fluorescence spectra at pH 8.0 under cryogenic conditions (Figures 6 and S1). The fluorescence intensity was significantly enhanced at cryogenic temperatures, while the excitation and emission bands remain around 437 and 573 nm, respectively (Figure S1). Peaks became sharper and exhibited a slight blue-shift (566 nm at 78 K), possibly due to a narrower vibrational energy distribution and reduced solvation dynamics in the S1 state (Figure 6A). Nevertheless, the strong orange emission at 78 K indicates that the high ESPT efficiency was retained under cryogenic conditions.

**Figure 6:**
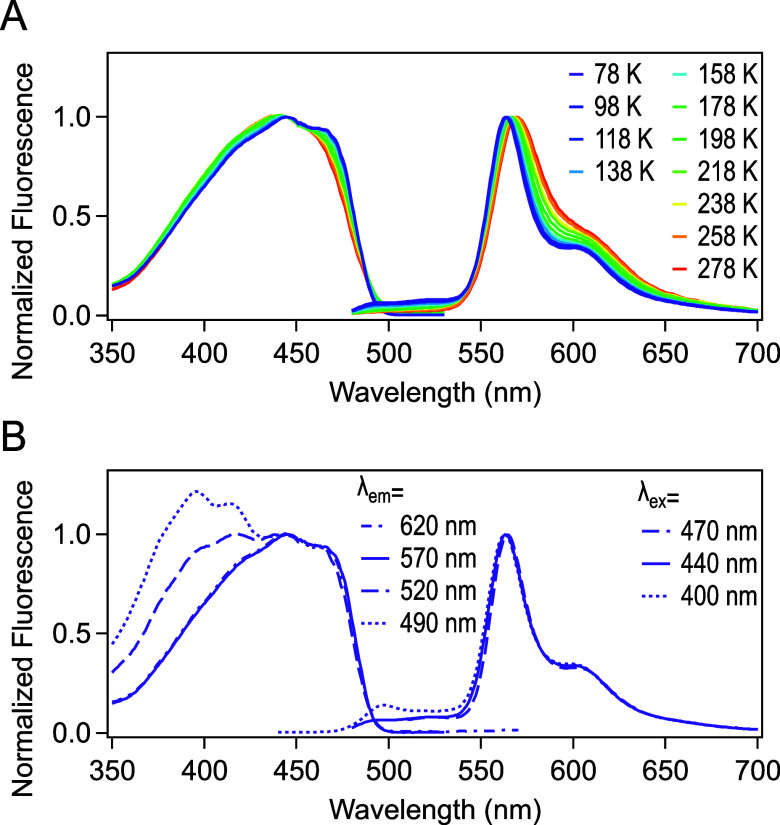
Normalized fluorescence spectra of LSSmOrange at pH 8.0 under cryogenic conditions. (A) Peak-normalized excitation (left) and emission (right) spectra at various temperatures. The excitation spectra were recorded with an emission wavelength of 570 nm. The emission spectra were recorded using excitation at 440 nm. LSSmOrange in solution (64 μM) in 50 mM HEPES buffer (pH 8.0) was used for the measurement. (B) Peak-normalized fluorescence spectra at 78 K. Excitation spectra were recorded with an emission wavelength of 490 nm (dotted line), 520 nm (dashed line), 570 nm (solid line), or 620 nm (chained line). Emission spectra were recorded using excitation at 400 nm (dotted line), 440 nm (solid line), or 470 nm (dashed line). Unnormalized spectra are shown in Figure S1.

The excitation spectra measured at lower temperatures were more structured and showed energy spacing between different vibrational levels (Figure 6A). The emission spectrum displays more defined vibration bands (566 and 604 nm) at 78 K, whereas at RT, these bands are more entangled. As the excitation profiles detected at 570 and 620 nm were similar, these two main peaks likely correspond to vibronic bands arising from excitation of the same species (Figure 6B).

In addition, two small emission peaks around 500 nm were observed at 78 K, which were not detected at RT, most likely due to a reduced internal conversion (Figure 6A). These two peaks were situated in the range of the emission band of the doubly protonated state detected at pH 3.0. Moreover, the excitation spectra measured at 520 and 490 nm were broadened toward shorter wavelengths (Figure 6B). The green emission detected around 500 nm in the spectra possibly originate from the doubly protonated species that might be present as a small fraction of the chromophore population at pH 8.0.

### Crystal Structure of Neutral State LSSmOrange under a Cryogenic Condition

The earlier reported structure of LSSmOrange (PDB: 4Q7R) has been determined at cryogenic temperature using a crystal grown at pH 4.5.^6^ Given the p*K*_a_ values of LSSmOrange (vide supra), it was reasonable to assume that this structure mainly reflected the doubly protonated state of the protein. With the aim of acquiring the crystal structure of LSSmOrange in the neutral (=singly protonated) state, we screened for conditions to grow microcrystals at near neutral pH. We obtained uniform needle-shaped microcrystals (10–30 μm in length; Figure S2B) at pH 8.0, from which a 1.9 Å resolution data set was collected at the BL41XU beamline of the SPring-8 synchrotron, using conventional oscillation-based crystallography at 100 K (cryo-X-ray crystallography). The crystals were characterized by the monoclinic space group *P*2_1_ with the following unit cell parameters: *a* = 39.27 Å, *b* = 85.05 Å, *c* = 41.71 Å, and β = 116.2°. There was one LSSmOrange molecule in the asymmetric unit. The structure was solved by molecular replacement using PDB entry 4Q7R as a starting model (Table 1).

**Table 1:** Crystallographic Analysis and Refinement Statistics for LSSmOrange at pH 8.0

	cryo-X-ray crystallography	RT SFX	pump–probe TR-SFX (50 μJ laser with 250 ps delay)
PDB entry	9LD5	9LD8	9LD9
Data Collection			
space group	*P* 1 2_1_ 1	*P* 1 2_1_ 1	*P* 1 2_1_ 1
unit cell *a*, *b*, *c* (Å)	39.27, 85.05, 41.71	38.50, 85.90, 40.00	38.50, 85.90, 40.00
α, β, γ (deg)	90.0, 116.2, 90.0	90.0, 110.8, 90.0	90.0, 110.8, 90.0
X-ray source	Spring-8 BL41XU	SACLA XFEL BL3(EH2)	SACLA XFEL BL3(EH2)
wavelength (Å)	1.00	1.23	1.23
resolution range (Å)	37.42–1.90 (2.02–1.90)^a^	33.22–1.70 (1.72–1.70)	33.22–2.00 (2.03–2.00)
hit images	N/A	122,512	40,831
indexed images	N/A	101,091	37,331
total reflections	530,091 (82,184)	33,462,104 (1,190,850)	8,854,501 (311,483)
unique reflections	19,429 (3256)	26,773 (1339)	16,473 (799)
multiplicity	25.2 (25.2)	1249.8(889.4)	537.5(389.8)
completeness (%)	99.94 (99.90)	100.0 (100.0)	100.0 (100.0)
*I*/σ(*I*)	10.04 (1.14)	9.42 (0.91)	8.25 (2.69)
Wilson *B*-factor (Å^2^)	21.5	39.9	36.0
*R*_meas_ (%)	61.6 (2594.5)	N/A	N/A
*R*_split_ (%)	N/A	6.4 (136.30)	8.4 (39.40)
CC_1/2_	99.5 (0.645)	0.995 (0.421)	0.989 (0.842)
Refinement			
refinement type	classical	classical	extrapolated
resolution range in refinement	37.42–1.9 (1.968–1.9)	28.2–1.7 (1.761–1.7)	28.2–2.0 (2.071–2.0)
number of reflections used in refinement	19,395	26,736	16,435
number of reflections used for *R*_free_	965 (94)	1341 (136)	824 (81)
*R*_work_ (%)	0.1797 (0.2753)	0.1632 (0.6314)	0.3003 (0.3802)
*R*_free_ (%)	0.2057 (0.3340)	0.2023 (0.6298)	0.3783 (0.4948)
number of non-hydrogen atoms	2092	2195	2109
macromolecules	1879	1957	1831
ligands	37	33	33
solvent	176	205	79
number of protein residues	230	231	226
RMSD bond length (Å)	0.006	0.004	0.003
RMSD bond angle (deg)	0.93	0.77	0.65
Ramachandran plot			
outliers (%)	0.00	0.44	0.00
allowed (%)	2.65	2.20	3.18
favored (%)	97.35	97.36	96.82
rotamer outliers (%)	0.00	0.00	1.06
clash score	2.38	0.77	1.38
average *B*-factor (Å^2^)	27.5	36.9	21.0
macromolecules	27.2	35.7	20.9
ligands	25.4	27.2	15.4
solvent	31.3	50.7	24.4

a Statistics for the highest resolution shell are shown between parentheses. N/A; not applicable.

The structure of LSSmOrange at pH 8.0 had the typical overall structure of a fluorescent protein, namely, an 11-stranded β-barrel that surrounds the chromophore (Figure 7A). The electron density around the residues composing the chromophore, T66-Y67-G68, was fitted to the reported chemical structure 2-[(4-)-5-methyl-2-hydroxy-3-oxazoline]-4-(*p*-hydroxybenzylidene)-5-imidazolinone (Figure 7B). The hydroxybenzylidene moiety was in a *cis* configuration. This chromophore structure at pH 8.0 overlapped well with the reported structure at pH 4.5 (RMSD 0.194 Å) (Figure 7C). The hydroxyl oxygen of S146 was located close to the chromophore hydroxyl oxygen (3.0 Å) and the D161 carboxylate oxygen (Oδ2, 2.6 Å), indicating the formation of a hydrogen bond network. The E215 carboxylate group, proposed to undergo Kolbe decarboxylation during photoconversion, was observed 4.2 Å away from the nitrogen atom (N2) of the imidazolinone moiety, excluding the formation of a direct hydrogen bond between the chromophore and E215. Reduced electron density was observed for the E215 carboxylate group in the 2m*F*_obs_–D*F*_calc_ map (Figure 7F), which could indicate specific X-ray radiation damage. The average dose (exposed region) for the data set was 9.93 MGy, which complied with the beamline’s guidelines and was three times lower than the dose above which biological information could be compromised in cryo-cooled structures.^12^ Nevertheless, the E215 carboxylate group might be highly radiation-sensitive due to its proximity to the electrophilic chromophore.

**Figure 7:**
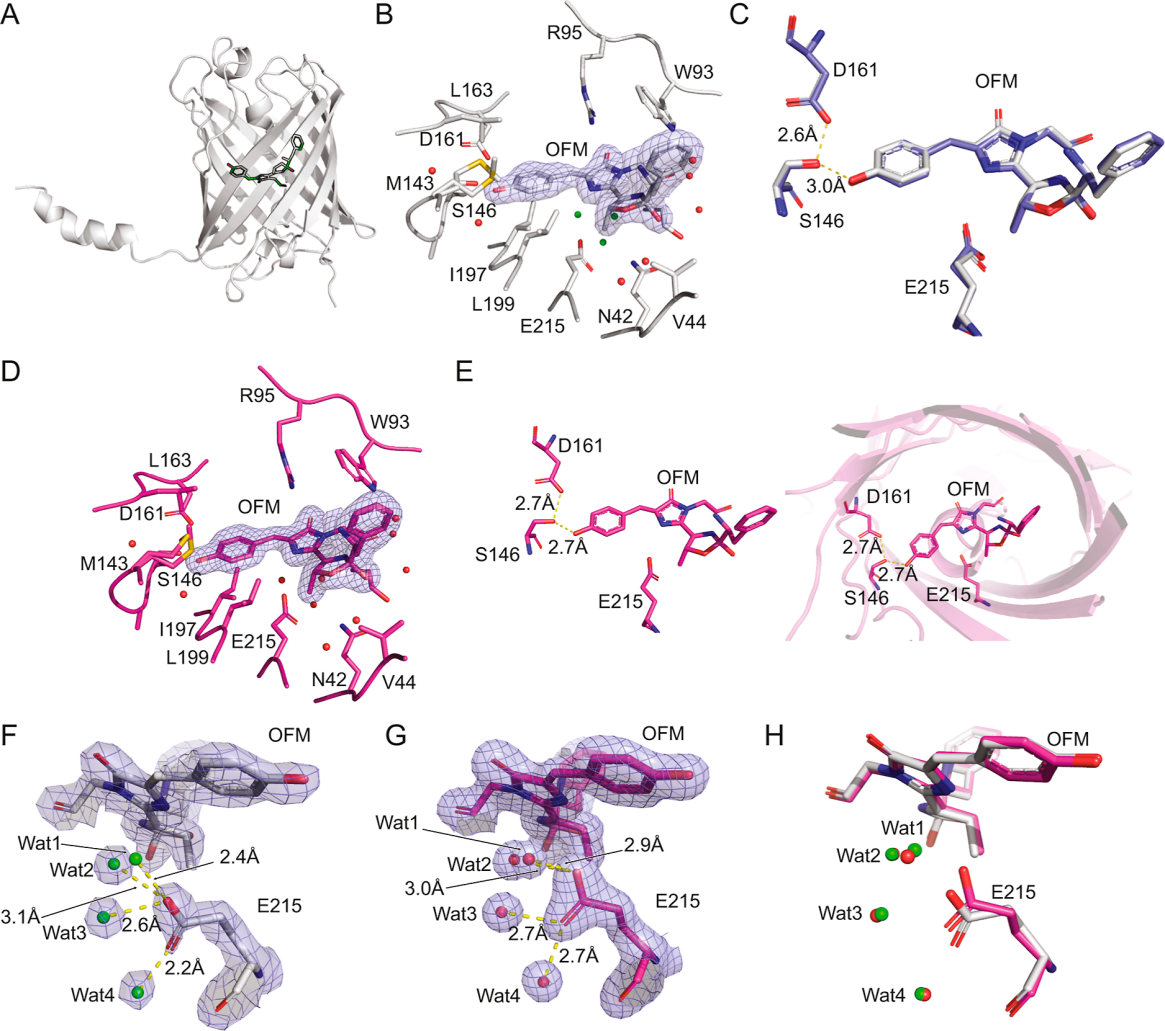
Crystal structures of LSSmOrange solved by cryo-X-ray crystallography and RT SFX. (A) Overall structure of LSSmOrange based on cryo-X-ray crystallography. The chromophore is highlighted by the black outline. (B) The chromophore pocket structure determined by cryo-X-ray crystallography. The 2m*F*_obs_–D*F*_calc_ map around the chromophore is contoured at 1.0 r.m.s.d. Water molecules interacting with E215 are shown in green. (C) Superimposed structures of the chromophore, S146, D161, and E215 from the cryo-X-ray crystallography (pH 8.0, white) data and 4Q7R (pH 4.5, purple). (D) The chromophore pocket structure based on RT SFX data. The 2m*F*_obs_–D*F*_calc_ map around the chromophore is contoured at 1.0 r.m.s.d. (E) (Left) The hydrogen bond network between the chromophore, S146 and D161 of the RT SFX structure. (Right) The position of the residues shown in the left panel with respect to the β-barrel. (F) Hydrogen bonds formed between E215 and nearby water molecules in the structure determined by cryo-X-ray crystallography. (2m*F*_obs_–D*F*_calc_ map contoured at 1.5 r.m.s.d.). (G) Hydrogen bonds formed between E215 and nearby water molecules in the structure determined by RT SFX. (2m*F*_obs_–D*F*_calc_ map contoured at 1.5 r.m.s.d.). (H) Overlay of the structures shown in (F) (structure in white, water molecules in green) and (G) (structure in magenta, water molecules in red).

### Structure of LSSmOrange Determined at RT by SFX

The fluorescence spectra of LSSmOrange at pH 8.0 differed significantly between cryogenic temperatures and RT (Figure 6). Therefore, we attempted to determine the LSSmOrange structure at RT. Diffraction data were collected by SFX at the BL3­(EH2) beamline of the SACLA XFEL, using 10 keV, <10 fs X-ray pulses at a repetition rate of 30 Hz. The microcrystals were embedded in hydroxyethyl cellulose^13^ and were delivered to the X-ray beam by a high-viscosity extrusion (HVE) system.^14^ In total, 101,091 diffraction patterns were indexed, which enabled the merge of a 1.7 Å data set. The crystals belonged to the same space group (*P*2_1_) as the cryo-X-ray structure (pH 8.0, 100 K), but differences in the unit cell parameters were observed. Notably, the β angle increased from 110.8° to 116.9° upon flash cooling. Additionally, the lengths of the axes also changed: from 38.50 to 39.27 Å (*a*-axis), from 85.90 to 85.05 Å (*b*-axis), and from 40.00 to 41.71 Å (*c*-axis). Overall, the unit cell is ∼1.1% more compact at RT than at 100 K, with unit cell volumes of 123,664 and 124,995 Å^3^, respectively. This observation is counterintuitive and uncommon; flash cooling of macromolecular crystals to 100 K is typically associated with a 3.5–5% reduction in unit cell volume.^15^

The RT SFX structure of LSSmOrange closely resembled the cryo-X-ray structure at 100 K (Figure 7D). The distance from the S146 hydroxyl oxygen to both the chromophore hydroxyl oxygen and the D161 carboxylate oxygen (Oδ2) was 2.7 Å (Figure 7E). These distances were 0.3 and 0.1 Å longer, respectively, than the corresponding distances at 100 K (Figure 7C). The carboxylate group of E215 was closer to the chromophore at RT: the distance between the imidazolinone N2 and E215 Oε2 was 3.5 Å, which was 0.7 Å shorter than in the structure at 100 K. Notably, the electron density of the E215 carboxylate group was much better defined in the SFX structure than in the cryo-X-ray structure (Figure 7F,G), most likely thanks to the continuous delivery of fresh crystals to the beam spot and the acquisition of diffraction patterns using extremely short XFEL pulses (diffraction before destruction).^16^ This supports the hypothesis that this residue undergoes specific radiation damage by synchrotron radiation during data collection.

Another major difference was observed in the water network around the chromophore, which was better defined and more connected in the SFX structure compared to the cryo-X-ray structure (Figure 7H). In brief, four water molecules were observed around the E215 carboxylate group in the SFX structure. Wat1 and Wat2 formed hydrogen bonds with E215 Oε2, at distances of 2.9 Å and 3.0 Å with O_Wat1/2_-Oε2-Cδ angles of 115° and 110°, respectively (Figure 7G). Wat3 and Wat4 formed hydrogen bonds with Oε1, each at a distance of 2.7 Å, with O_Wat3/4_-Oε1-Cδ angles of 121° and 155°, respectively. In the cryo-X-ray structure, however, Wat1 showed reduced electron density, and the O_Wat1_-Oε2-Cδ increased to 176°, which is too large to form a hydrogen bond (Figure 7F). Moreover, the altered conformation of the E215 carboxylate group caused Wat3 to interact with E215 Oε2 (2.6 Å, O_Wat3_-Oε2-Cδ angle of 112°) in the cryo-X-ray structure instead of with Oε1, as observed in the SFX structure. The absence of the second hydrogen bond to E215 Oε1 seemed to be compensated by a stronger hydrogen bond to Wat4 with a shorter distance of 2.2 Å, while maintaining a similar angle (152° vs 155°). Thus, only the interaction between Wat2 and E215 Oε2 was preserved in both structures (3.1 Å, an O_Wat2_-Oε2-Cδ angle of 118° in the cryo-X-ray structure).

### Time-Resolved Analysis of LSSmOrange Structure by Pump–Probe TR-SFX

The decarboxylation of E215 has been reported as the key reaction for photoconversion in LSSmOrange.^4^ The absence of radiation damage to the carboxylate group of E215 in the SFX structure prompted us to conduct an optical pump—X-ray probe TR-SFX experiment to investigate the structural dynamics of the photoconversion process. A circularly polarized 400 nm second harmonic produced by a 50 fs Ti/sapphire laser (800 nm) was used to excite LSSmOrange (pump). We employed a circularly polarized laser beam to maximize the efficiency in excitation of crystals that arrive in the focal plane in different orientations. The pump laser was focused onto the crystals with a spot diameter of 85 μm (FWHM), yielding a mean fluence of 0.31 J/cm^2^ and an average power density of 6.3 TW/cm^2^ (Table 2). This exceptionally high pump-laser fluence, which corresponded to a σ F/hν of 124, was chosen by taking the extremely low photoconversion quantum yield associated with single-photon excitation into account. The choice of the appropriate pump-laser fluence during a TR-SFX experiments to study physiological structural dynamics, especially to minimize the impact of multiphoton absorption, is currently under debate.^17^ However, in this study, the multiphoton absorption resulting from the high laser power apparently facilitated efficient photoconversion, which was essential for the analysis by TR-SFX. Our time-resolved spectroscopic experiments further confirmed that multiphoton absorption indeed promoted efficient photoconversion (vide infra).

**Table 2:** Optical Pump Laser Parameters for TR-SFX

pump energy (μJ)	50
pump wavelength (nm)	400
focal diameter FWHM (μm)	85
mean fluence through FWHM (J/cm^2^)^a^ ^,^ ^b^	0.31
pulse duration (fs)	50
average power through FWHM (TW/cm^2^)	6.3
molar absorption coefficient (M^–1^ cm^–1^)	5.2 × 10^4^
protein concentration (mM)	27
absorption cross section (cm^2^)	2.00 × 10^–16^
photon energy at 400 nm (J)	4.97 × 10^–19^
σ F/hν (photons)	124
1/e depth (μm)	3.1
1/10 depth (μm)	7.1

a Energy in FWHM is 70% of the total pump energy.

b Half of the energy was lost by polarizing the beam circularly.

TR-SFX data were collected by HVE injection of hydroxyethyl cellulose embedded microcrystals, using XFEL pulse (SACLA BL3­(EH2), 10 keV, <10 fs) at a repetition rate of 30 Hz and a pump–probe delay of 250 ps. Diffraction patterns with and without pump laser illumination were acquired in an interleaved fashion with 0.38 mm distance between consecutive shots on the viscous jet (linear flow velocity of 5.7 mm/s and pump laser repetition frequency of 15 Hz). The absence of “light contamination” with this spacing was confirmed by computing a Bayesian-weighted difference Fourier electron density map between the dark data set acquired without the pump laser illumination (“complete-dark”) and the data set consisting of images collected without pump laser illumination during the interleaved acquisition mode (“interleaved-dark”) (*F*_obs,_^interleaved‑dark^–*F*_obs,_^complete‑dark^; Figure S3). Thus, both sets of dark images were merged into a single all-dark data set to rerefine the RT-SFX dark state structure (Figure S4) and to analyze the phototriggered structural changes. Changes in electron density upon excitation with the pump laser were visualized by the computation of a Bayesian-weighted difference Fourier electron density map (*F*_obs,_^light‑250ps^–*F*_obs,_^all‑dark^; Figure 8A,B).^18^ Strong negative difference density at the E215 carboxylate group was observed, supporting the hypothesis that this residue undergoes decarboxylation. No significant difference density was present on the chromophore.

**Figure 8:**
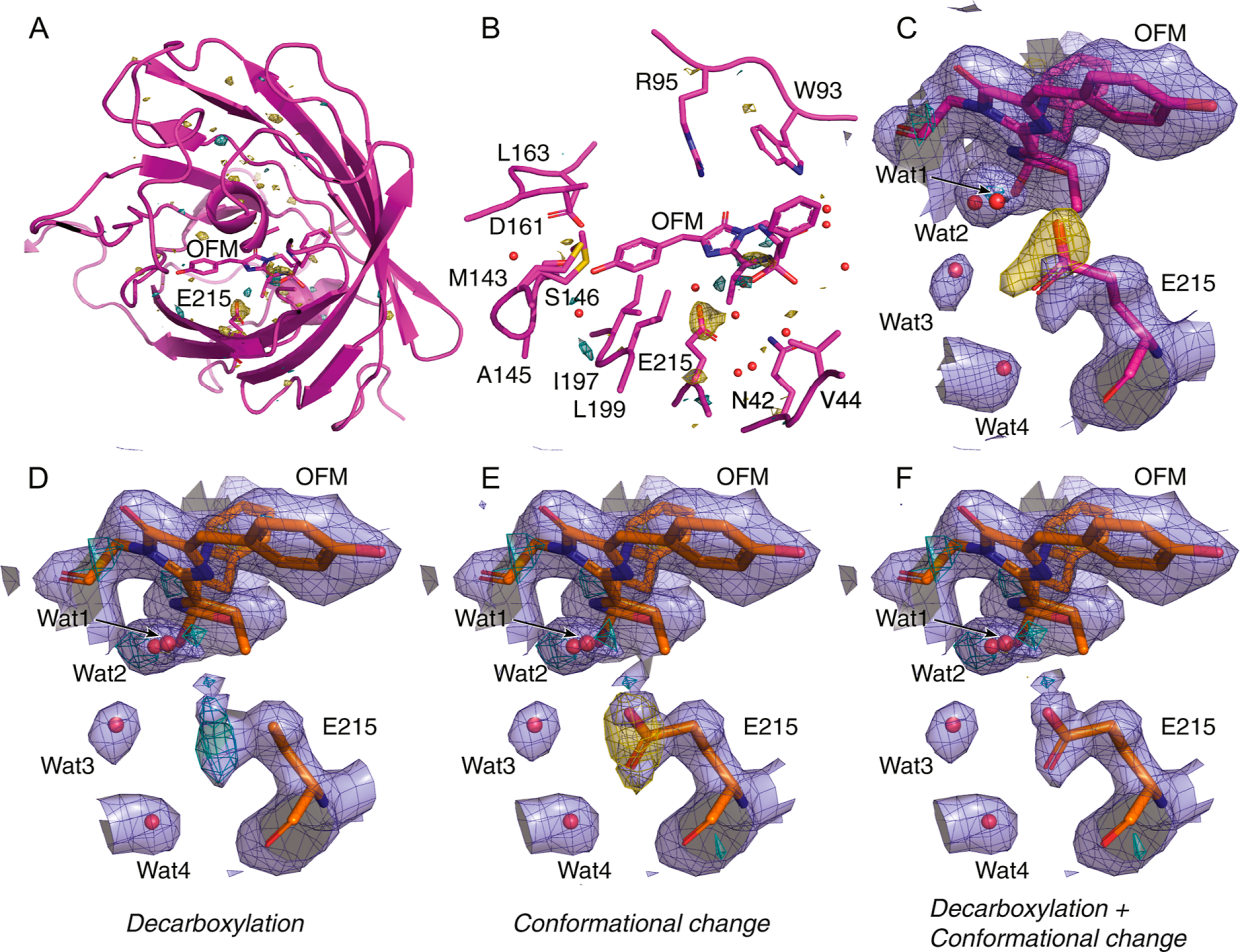
Pump–probe TR-SFX shows decarboxylation and conformational change of E215. (A) *F*_obs,_^light‑250ps^–*F*_obs,_^all‑dark^ Fourier difference map over the full protein. The long N-terminus was omitted from the figure for clarity. Map contour level: ±4 r.m.s.d.; teal: positive electron density; yellow: negative electron density; magenta: dark state structure. (B) Same as (A) but zoom on the chromophore pocket. (C) Extrapolated maps calculated with an occupancy of 12% and phases of the dark state model. Blue: 2m*F*_extr_–D*F*_calc_, contoured at 1 r.m.s.d.; teal/yellow m*F*_extr_–D*F*_calc_ contoured at ± 4 r.m.s.d.; magenta: dark state structure. (D–F) Refinement of the 250 ps-light adapted structure in extrapolated structure factor amplitudes. Refinement with a decarboxylated E215 leads to significant remaining positive extrapolated difference electron density around E215 (D). Refinement with a single altered E215 conformation leads to strong negative extrapolated difference electron density on the E215 carboxylate group (E). Refinement with partial decarboxylation and partial altered conformation of E215 (with a relative occupancy of 54 and 46%, respectively) fits the extrapolated electron density well (F). Color codes of models and maps in panel (D,E,F): blue: refined 2m*F*_extr_–D*F*_calc_, 1 r.m.s.d.; teal/yellow: refined m*F*_extr_–D*F*_calc_ contoured at ± 3 r.m.s.d.; orange: refined 250 ps model with E215 as indicated below each panel.

Aside from this observed negative density at E215, minimum structural changes were observed in the overall structure of the protein during the pump–probe TR-SFX experiment (Figure 8A,B). This observation highlights the structural rigidity of the protein backbone within the 250 ps time scale of the experiment. After ESPT, which occurs in the order of ps (0.8 ps),^4^ LSSmOrange emits orange fluorescence. Therefore, the structure 250 ps after excitation corresponds predominantly to that of unconverted anionic LSSmOrange that is formed as a result of the ESPT process. The absence of conformational changes associated with the accumulation of the anionic species suggests that the ESPT process involves minimal structural changes. This hypothesis is supported by the observation that ESPT is the dominant relaxation pathway even at 78 K, where molecular motions are frozen, while proton movement within the hydrogen bond network involved in the ESPT pathway still can occur.

Recently, a photoenzyme that converts fatty acids into alkanes or alkenes, fatty acid photodecarboxylase (FAP), was identified in the alga *Chlorella variabilis*.^19^ The flavin adenine dinucleotide (FAD) functions as a cofactor of FAP, and upon photoexcitation, FAD accepts an electron from the substrate’s carboxylate group to trigger Kolbe-type decarboxylation. This mechanism is analogous to the decarboxylation mechanism we proposed for LSSmOrange, in which the excited chromophore accepts an electron from the carboxylate group of E215. In the case of FAP, the formation of carbon dioxide (∼270 ps) occurred concomitantly with the decay of ^1^FAD* (∼300 ps).^20^ The photoinduced decarboxylation by FAP was also confirmed by TR-SFX experiments, in which substantial negative electron density corresponding to the decarboxylation was observed at 900 ps, consistent with the kinetics of ^1^FAD* decay and carbon dioxide formation. We observed decarboxylation of LSSmOrange at 250 ps by TR-SFX, which is a time scale comparable to that of FAP. This similarity is consistent with our proposed mechanism of LSSmOrange involving electron transfer to the photoactivated chromophore. Similar decarboxylation reaction via electron transfer to a chromophore has also been reported in lactate monooxygenase (LMO).^21,22^ LMO catalyzes the decarboxylation of lactate using flavin mononucleotide (FMN) as an electron acceptor, which is followed by oxidation to produce acetate. In LMO, electron transfer from the substrate’s carboxylate group to FMN (forward ET) typically proceeds thermally, but photoinduced electron transfer was recently reported, described as “unnatural photodecarboxylation”. The photoinduced forward ET occurred within 1.5 ps, whereas electron transfer back to the substrate also occurred rapidly (1.1 ps), greatly impacting the catalytic efficiency. We have analyzed the excited-state kinetics of LSSmOrange by femtosecond time-resolved spectroscopy,^4^ but the reduced form of chromophore was not observable under our experimental conditions, preventing detailed kinetic analysis. Further kinetic analysis of the excited species of LSSmOrange remains to be performed.

### Bayesian-Weighted Extrapolated Structural Model

Structural changes taking place 250 ps after pump laser illumination were modeled and refined on the basis of Bayesian-weighted extrapolated structure factor amplitudes and related maps (Figure 8C).^18^ The estimated occupancy of the light-adapted state in the crystals ranged from 12 to 16%. Guided by the observation of a negative peak on the E215 carboxylate group in the *F*_obs,_^light–250ps^–*F*_obs,_^all‑dark^ map and the reduced electron density for its atoms in the extrapolated map, we first produced and refined a model with a decarboxylated E215 side chain. However, this was insufficient to fit the extrapolated 2m*F*_ext_–D*F*_calc_ map as clear electron density was still visible for the E215 side chain (Figure 8D). Furthermore, refinement of the model led to the appearance of a positive peak in the refined difference map (m*F*_ext_–D*F*_calc_). Therefore, we tested the possibility that a part of the released carbon dioxide still resides in the chromophore pocket, but modeling a free carbon dioxide molecule was difficult due to the significant overlap with the remaining E215 side chain (Cγ) after the decarboxylation (Figure S5). We then tested whether E215 adopts an alternative conformation upon light illumination and found that an additional E215 conformer with an altered side-chain conformation fits the electron density well. However, this conformer alone was also not sufficient to accurately describe the data, as indicated by the appearance of a negative peak in the refined difference maps (m*F*_ext_–D*F*_calc_) (Figure 8E). This motivated us to refine a model in which the E215 side chain is partly decarboxylated and partly in a different conformation, which resulted in the best fit in the extrapolated electron density (Figure 8F) and no m*F*_ext_–D*F*_calc_ difference peaks on the E215 side chain. Thus, we propose that our structural data provide insights into a mixture of two states, in which E215 is either partly decarboxylated or adopts an alternate conformation. Furthermore, no electron density corresponding to released carbon dioxide was observed within the chromophore pocket in the difference or extrapolated maps. This suggests that the carbon dioxide does not adopt a well-defined, crystallographically resolvable position.

In addition, a small backbone shift was observed in the 250 ps structure, which led to a slightly more compact β-barrel than in the dark-state, with an overall Cα superposition RMSD of 0.2 Å (for comparison, the RMSD value between the structures refined against the “interleaved-darks” and “complete-dark” data sets was only 0.02 Å) (Figure S6).

### Nonlinear Energy Dependency of LSSmOrange Photoconversion

Our TR-SFX experiment revealed the decrease in electron density at the E215 carboxylate group upon the 400 nm pump laser illumination, consistent with Kolbe-type decarboxylation, a key aspect of LSSmOrange photoconversion.^4^ However, the reported quantum yield of this process is extremely low (8.5 × 10^–6^),^5^ raising the question why such a low quantum yield reaction could be observed by TR-SFX. We noticed a crucial difference in the illumination mode between our TR-SFX experiment and the one used to determine the reported quantum yield. In the latter, LSSmOrange was illuminated with a continuous-wave (CW) laser emitting at 458 nm with a power density of 30.2 kW/cm^2^ or lower. This was 8 orders of magnitude lower than the average power density within the FWHM of the optical pump laser used during the TR-SFX experiment (6.3 TW/cm^2^). Such a high power density corresponded to 124 photons passing through the (one-photon) absorption cross section at the crystal surface (σF/hν). Thus, we hypothesized that nonlinear optical processes occurring in crystals were responsible for the observed photoconversion in our TR-SFX experiment.

To test this hypothesis, we developed a spectroscopic system to evaluate the photoconversion efficiency. In this system, LSSmOrange molecules in solution were photoconverted by focused 400 nm femtosecond laser pulses with a repetition rate of 1 kHz. In each 1 ms illumination cycle (demarcated by the photoconversion pulses), the accumulation of the photoconverted species was monitored by detecting transient absorption (|ΔOD|) 1 ns before the start of the next cycle. For detection, a 520 nm laser was used to excite the photoconverted species. The transient absorption signal at 560 nm (a combination of ground-state depletion and stimulated emission) was then recorded 5 ps after excitation. Since only the photoconverted species was excited under this condition, the transient absorption signal reflected the amount of photoconverted LSSmOrange species that had accumulated in the focal volume of the measurement.

The transient absorption signal (|ΔOD|) increased immediately after turning the 400 nm laser on, reaching a steady state within a few minutes (Figures 9 and S8). The steady state was established as a balance among the photoconversion rate, the diffusional efflux of the photoconverted species from the observation volume, and the diffusional influx of the unconverted species. As a result, the steady-state transient absorption signal was described as a function of the laser power using the following equation (see Text S1 for details)

$$\left(\left|{\Delta OD}_{ss}\right|-\left|{\Delta OD}_{off}\right|\right)=A_{1}\left(1-\frac{1}{A_{2}E^{n}+1}\right)$$

where *A*_1_ and *A*_2_ are constants that reflect the diffusion coefficients of both unconverted and converted species as well as the size and shape of the observation volume. In this equation, *n* = 1 is expected for a single-photon process, whereas *n* > 1 indicates a reaction induced by multiphoton absorption. The steady-state levels of transient absorption were measured with (|ΔOD_ss_|) and without (|ΔOD_off_|) illumination by the 400 nm photoconversion laser at various energies ranging from 0.1 to 1.0 μJ and were fitted to the function above. The steady-state transient absorption signals fit the function well with an *n* value of 2.97 ± 0.50, which implied the involvement of a multiphoton absorption process in photoconversion. We thus concluded that photoconversion was induced via a multiphoton absorption process.

**Figure 9:**
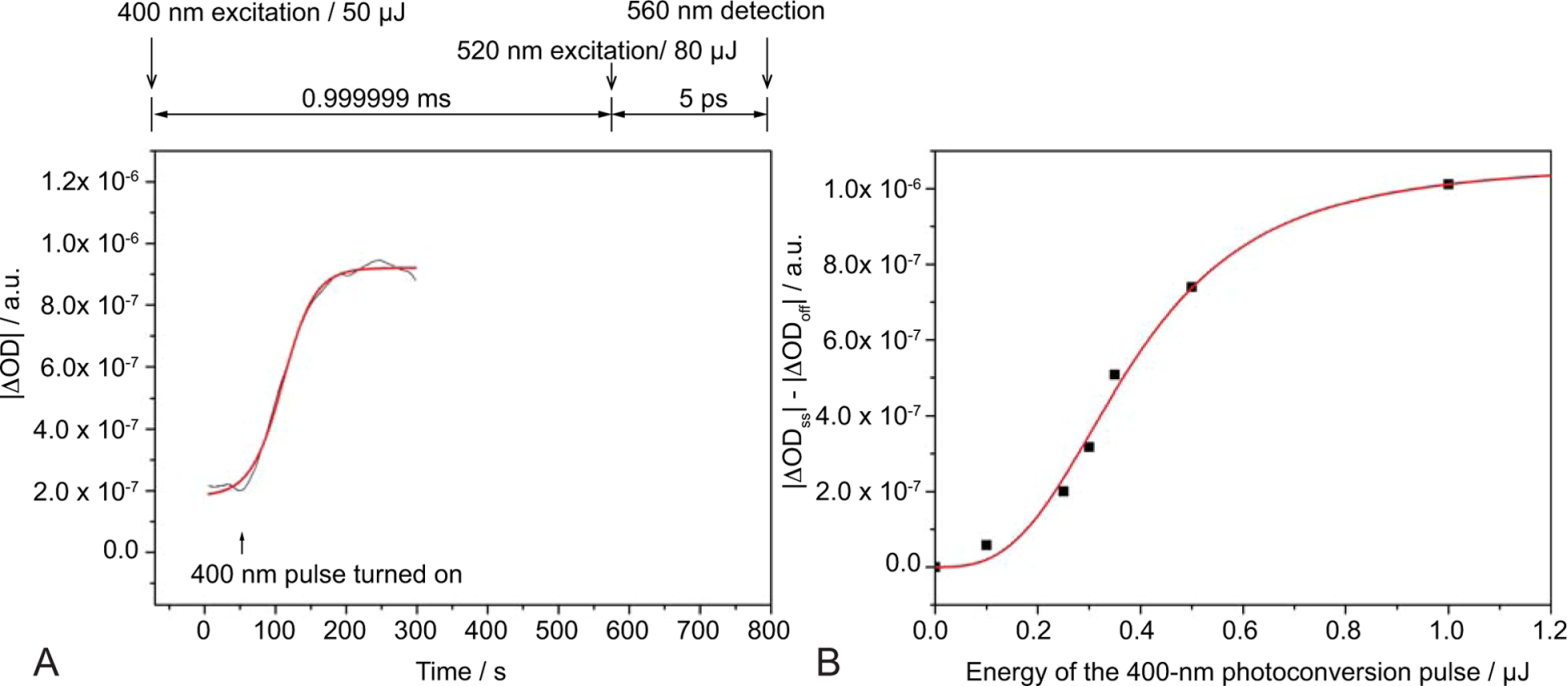
Enhanced LSSmOrange photoconversion via multiphoton absorption process. (A) Accumulation of photoconverted LSSmOrange by repetitive illumination with a 100 fs pulse laser at 400 nm. The repetition rate of the photoconversion laser was 1 kHz. The amount of photoconverted LSSmOrange was estimated by exciting the photoconverted species with a 520 nm pump laser and by detecting the combination of the ground-state absorption and stimulated emission signals at 560 nm (|ΔOD|). The result with a pulse energy of 0.5 μJ is shown as a representative. The measurement was repeated with various pulse energies ranging from 0.1 μJ to 1.0 μJ (Figure S8). (B) Nonlinear susceptibility of LSSmOrange. The photoconversion rate was evaluated from the accumulated LSSmOrange at the steady state (|ΔOD_ss_|-|ΔOD_off_|). Regression analyses revealed that the correlation between the photoconversion rate and the pulse energy of the photoconversion laser followed the model of a nonlinear optical process with χ^2^ = 6.19 × 10^–14^.

Kolbe decarboxylation is triggered by the transfer of an electron from the E215 carboxylate group to the excited-state chromophore (Figure 10A). Consequently, the oxidizing power of the chromophore is essential to trigger the photoconversion reaction, and the oxidation potentials depend on the electronic excited states. Since 400 nm lies within the range of the main absorption band of the neutral chromophore of LSSmOrange, the transition to the S_1_ state is induced by single-photon absorption. In this situation, an electronic transition from the highest occupied molecular orbital (HOMO) to the lowest unoccupied molecular orbital (LUMO) is expected (transition from N to N*) (Figure 10C). The chromophore in the N* state serves as an oxidizing agent that can accept an electron in its singly occupied molecular orbital (SOMO). However, the oxidation power of the N* state chromophore is apparently insufficient to efficiently abstract an electron from the carboxylate group of E215 to evoke photoconversion. Upon illumination with a 400 nm femtosecond laser pulse, a transition to a higher-lying electronic excited state (S_n_) via a multiphoton process is assumed (Figure 10B). In theory, there are two possible pathways for the S_0_ to S_n_ transition (indicated as N_1_** and N_2_** in Figure 10C). One is a transition from a lower occupied molecular orbital (HOMO-n) to LUMO. As a result, the HOMO-n in the ground state becomes SOMO in the excited state (N_1_**). Thus, the SOMO is expected to have a lower energy level in the N_1_** state than in the N* state. The other pathway is an electronic transition from HOMO to a higher unoccupied molecular orbital (LUMO + *n*). In this case, the HOMO in the ground state becomes SOMO in the excited state (N_2_**), and hence the energy level of the SOMO in the N_2_** state is similar as that in the N* state. Considering the energy levels of the different excited state SOMOs, the highest oxidizing power is expected in the N_1_** state. Thus, we propose that the transition to the N_1_** state is the predominant pathway for the E215 decarboxylation associated with LSSmOrange photoconversion because of the high oxidizing power of this state.

**Figure 10:**
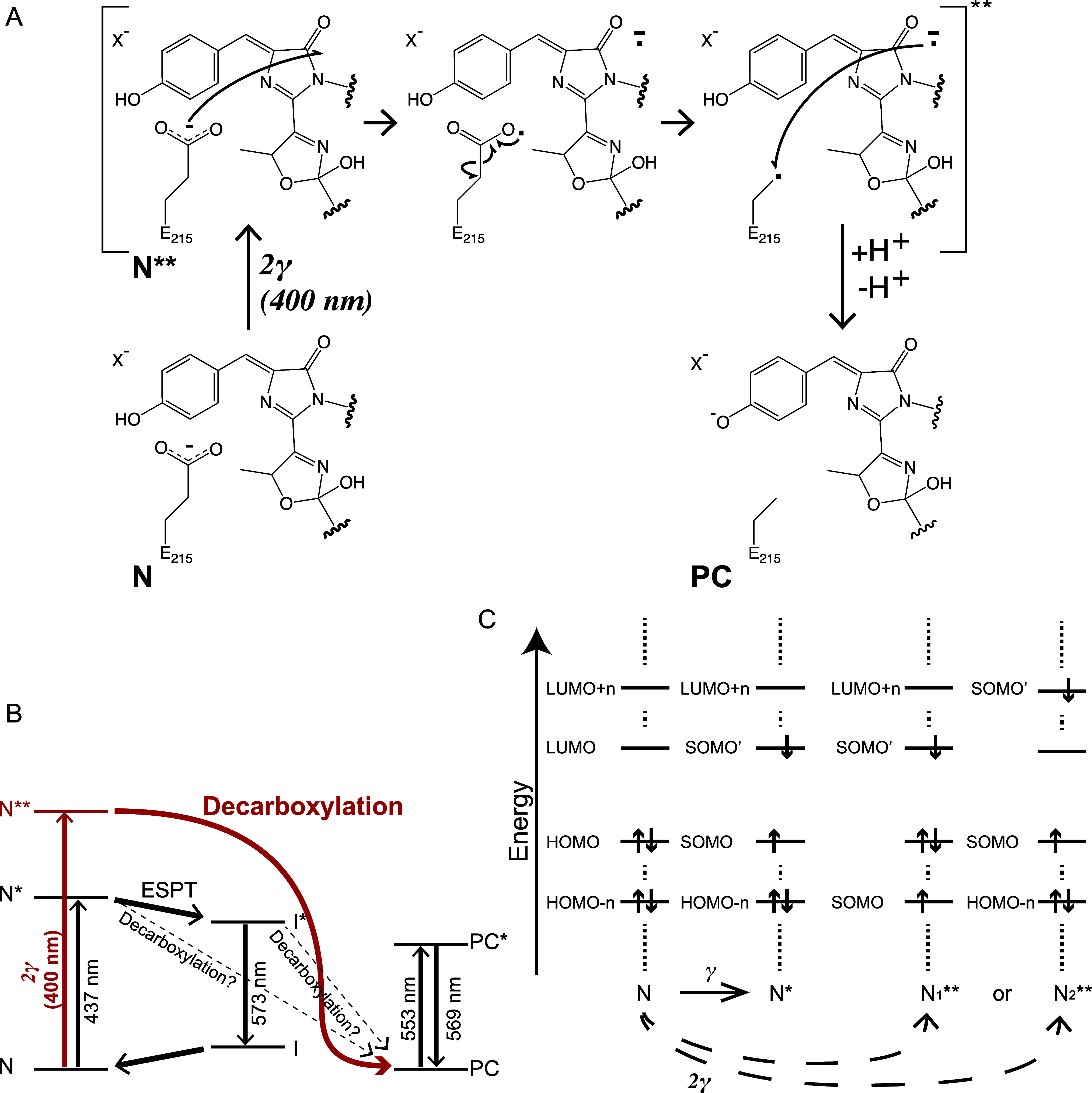
Proposed mechanism for E215 decarboxylation during photoconversion in LSSmOrange via multiphoton absorption. (A) Electron transfer pathway in the higher-lying excited state. The oxidation potential of the chromophore increases upon excitation to N** so it can accept an electron from the E215 carboxylate group, forming a carboxyl radical at this position, and triggering decarboxylation. The reaction at E215 is terminated by accepting an electron from the chromophore. (B) Excited-state processes of LSSmOrange. Photoconversion requires a higher-lying electronic excited state that is reached by a multiphoton (most likely two-photon) absorption process. (C) Energy diagram showing the possible excitation pathways of the N state chromophores. Single-photon absorption induces the transition to the S_1_ electronic exited state (N*). Since photoconversion is inefficient under the illumination with a continuum laser at around 440 nm, we suppose that the oxidation potential of the N* state is not high enough to efficiently abstract the electron from the E215 carboxylate group. Absorption of two photons results in the transition to a higher-lying electronic excited state (S_n_). This transition can either have the character of a transition of an electron from HOMO-n to LUMO (depicted as N_1_**) or from HOMO to LUMO + *n* (N_2_**). The resulting energy level of the SOMO is lower in the N_1_** than in the N* state, whereas the energy in the N_2_** state is similar to that in the N* state. Therefore, a higher oxidation potential than N* is only expected in the N_1_** state. We propose that the efficient electron transfer from the carboxylate group of E215 to the chromophore in the N_1_** state is the preferable pathway for the decarboxylation process associated with photoconversion.

Efficient Kolbe decarboxylation via a higher-lying electronic excited state may not be unique to the LSSmOrange. Bell et al. have shown efficient photoconversion of wild-type Aequorea GFP via one or more higher-lying electronic excited states (S_2_ or higher) using 254 nm illumination, whereas no photoconversion was detected upon 400 nm illumination.^23^ In addition, Langhojer et al. reported the efficient photoconversion of the T203 V mutant of Aequorea GFP via a two-photon process at 400 nm and a three-photon process at 800 nm.^24^ Moreover, they revealed that the 400 and 800 nm laser pulses evoked efficient photoconversion cooperatively. From this, they concluded that the singly excited neutral state is a resonant intermediate of the photoconversion of GFP via a two-step absorption.

Photoconversion of fluorescent proteins is a useful microscopic technique for highlighting specific protein molecules within defined volumes in living specimens, enabling the molecular tracking and monitoring of morphological changes in targeted cellular regions. Under a single-photon regime, however, spatial confinement of the photoconversion volume is challenging. While the laser is sharply focused on the focal plane, it also illuminates out-of-focus planes with a broader beam spot, where the photon density is lower but still sufficient to induce photoconversion. Consequently, photoconversion may occur in regions beyond the intended focal volume. Multiphoton regime may enable photoconversion within a more spatially confined volume. Previously, we demonstrated photoconversion of LSSmOrange using an 850 nm femtosecond pulse laser, both in vitro and in living cells.^5^ In our previous paper, we reported that the pulse laser induced confined photoconversion with an oval-shaped *yz*-profile of 2.0 ± 0.1 μm along the optical axis, in striking contrast to the double-cone-shaped photoconversion profile induced by a 458 nm CW laser. Furthermore, the pulse laser induced photoconversion within a confined region along the optical axis in living HeLa cells expressing LSSmOrange targeted into the mitochondria. We have described the observations as the result of the two-photon process, but the energy of the two-photon absorption process using an 850 nm pulse is equivalent to that of a 425 nm single photon, which is apparently insufficient to excite the molecule to higher-lying electronic states. Instead, the photoconversion with the 850 nm pulse may occur via a three-photon process, with an energy equivalent to that of a 283 nm single-photon process. Nevertheless, the combination of confocal imaging using CW lasers and multiphoton photoconversion with femtosecond pulse lasers expands the potential of LSSmOrange as a microscopic highlighter in living cells.

### Quantum-Chemical Calculations Support the Involvement of a Higher Electronic Excited State in Photoconversion

Quantum-chemical calculations were performed to investigate the orbital nature of the excited states lying closest in energy to the S_1_ transition which involves the HOMO and LUMO. These calculations, performed at the CAM-B3LYP­(D3BJ)/cc-pVDZ level of theory, employed a large cluster model (136 atoms) which included the chromophore, the side chain of E215, and surrounding residues, as described in the Supporting Information (Text S2 and Figure S9). Using this model, the lowest-lying excited state was predicted to be chromophore-centered with a predicted absorption wavelength of 373 nm (Table S1). Among the predicted higher-lying excited states, one had a dominant “charge-transfer” character involving electron transfer from the E215 carboxylate orbital to the chromophore LUMO with a predicted absorption wavelength of 286 nm (Figure 11). Unlike the S_1_ excited state, this state had a low transition dipole moment, suggesting that it is unlikely to be directly populated by the absorption from the ground state. Nevertheless, the fact that a state with an electron removed from the carboxylate group lies well below the energy that would be delivered by two 400 nm photons suggests that Kolbe-type decarboxylation of the E215 side chain likely involves a state of this type.

**Figure 11:**
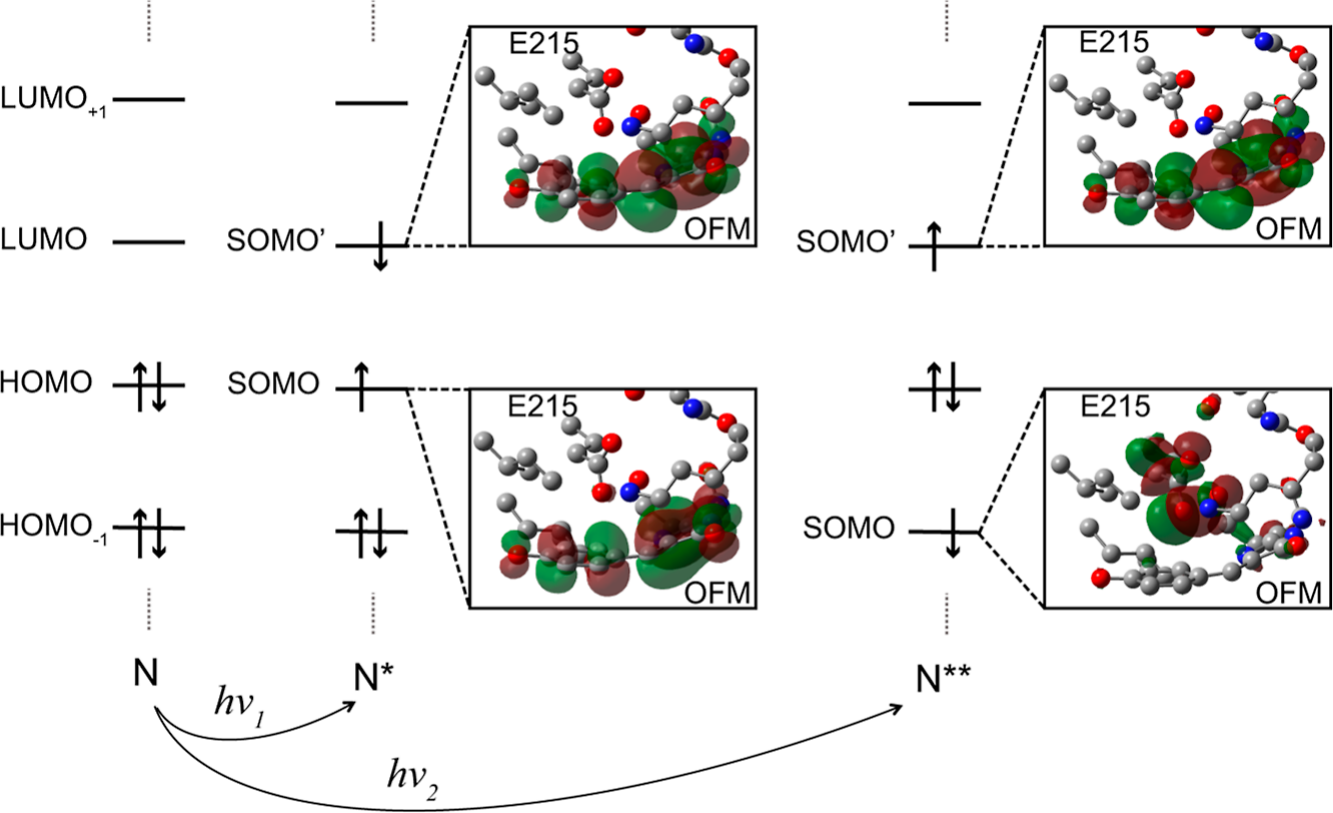
Computationally predicted electronic transitions for the S_1_ and higher excitations of LSSmOrange at the CAM-B3LYP­(D3BJ)/cc-pVDZ level of theory. The S_1_ excitation, N → N*, has a predicted absorption wavelength of 373 nm (*h*ν_1_). The higher-lying excitation, denoted N → N**, has 286 nm as predicted absorption wavelength (*h*ν_2_). The relevant natural transition orbital diagrams are given alongside each excitation process.

In separate work, we have used hybrid QM/MM methods on LSSmOrange and other fluorescent proteins, and these calculations confirm the mechanistic conclusions obtained here.^25^ Previous modeling studies have also found such charge-transfer states relevant to photoinduced decarboxylation of fluorescent protein GFP.^25–27^ Further work, including calculations of a larger number of excited states, evaluation of multiphoton absorption cross sections, and ideally excited-state molecular dynamics simulations, will be required to fully resolve the decarboxylation mechanism, particularly to characterize the nature of the local chromophore N_1_** excited state suggested in Figure 10.

## Conclusions

We observed the additional protonation of LSSmOrange under acidic conditions. The doubly protonated species exhibited extremely dim fluorescence and no detectable ESPT. Therefore, we investigated the structural dynamics of LSSmOrange at pH 8. SFX enabled us to determine the crystal structure of LSSmOrange at RT while avoiding detectable X-ray radiation damage, in accordance with the “diffraction before destruction” principle. TR-SFX revealed a decrease in electron density at E215, occurring 250 ps after excitation. This change was consistent with Kolbe decarboxylation, which is a key reaction for photoconversion. The photoconversion most likely proceeded via a multiphoton absorption process, facilitated by the exceptionally high energy density of the optical pump laser. Without high-energy illumination, the process might have remained undetectable by TR-SFX due to the extremely low photoconversion quantum yield of LSSmOrange. Furthermore, we demonstrated that the photoconversion process exhibited optical nonlinearity, requiring excitation to higher-lying electronic states, as evidenced by time-resolved spectroscopy and corroborated by quantum-chemical calculations. The efficient multiphoton-driven photoconversion of LSSmOrange enables precise molecular highlighting within a spatially confined region in live cells. Future advancements in microscopy, integrating multiphoton photoconversion with confocal imaging, will expand the potential applications of LSSmOrange as a high-precision optical highlighter.

## Materials and Methods

### Preparation of Recombinant LSSmOrange

The gene encoding LSSmOrange was amplified by PCR using LSSmOrange/pRSET as a template and was subcloned into the NcoI/XhoI site of pET28 (LSSmOrange/pET28). The ATG sequence in the NcoI site was used as the start codon of LSSmOrange, and the XhoI site was added in frame with the C-terminus. The added XhoI site encoded amino acid residues L and E, which served as a linker to connect the LSSmOrange to the His6-tag present in the pET28 vector. *E. coli* JM109­(DE3) cells were transformed with LSSmOrange/pET28 using kanamycin as a selection marker. Recombinant LSSmOrange was expressed in the transformed bacteria, extracted, and subjected to purification by metal affinity chromatography as previously described.^4^ LSSmOrange was further purified by size exclusion chromatography with a HiLoad 16/600 Superdex 75 preparative grade column using 20 mM HEPES pH 7.4 containing 150 mM NaCl as an elution buffer. Purified LSSmOrange was concentrated by ultrafiltration (Vivaspin Turbo 4 with a 10,000 molecular-weight cutoff). The buffer of the proteins was changed to 20 mM HEPES at pH 7.5 by using a PD-10 desalting column. The purity of the purified LSSmOrange proteins was checked by SDS-PAGE. The protein concentration was determined by measuring absorbance at 437 nm with NanoDrop 2000 (Thermo Scientific) using the extinction coefficient of 5.2 × 10^4^ M^–1^ cm^–1^.^3^

### Spectra Acquisition at RT

Absorption spectra were acquired with a spectrophotometer (NanoDrop 2000) in 50 mM citrate (pH 3.0–6.5), HEPES (pH 7.0–8.5), or glycine buffers (pH 9.0–11.0) containing 150 mM NaCl. Fluorescence spectra were acquired with a fluorofluorometer (Fluorolog-3, Horiba) equipped with an R928P photomultiplier tube detector and a 450 W xenon light source. Spectra data were analyzed using Igor Pro 7 software (WaveMetrics).

### Fluorescence Spectra under Cryogenic Conditions

A custom-made square glass cuvette was designed with approximate dimensions of 2 × 2 cm and a 0.5 mm spacer. The LSSmOrange solutions were diluted to 64 μM with 50 mM citrate buffer (pH 3.0) or HEPES buffer (pH 8.0) and loaded into a glass cuvette. The cuvette was sealed with epoxy glue (Epoxylijm gel, Sencys). A cryostat (JANIS VPF-100; Lake Shore Cryotonics) was employed to cool the sample. The sealed cuvette was placed in the cryostat between two gold plates of the optical sample holder. The sample was cooled to 78 K by filling the cryostat with liquid nitrogen. The temperature was controlled with a model 325 Temperature Controller (Lake Shore Cryotonics). The cryostat was set into a spectrofluorometer (FP-8550, Jasco), and the measurements were performed with 45° excitation to the sample surface and emission detected at 90° relative to the excitation. For emission spectra, the entrance slit was set to 2.5 nm (pH 8.0) or 5 nm (pH 3.0) and the exit slit to 1 nm. For excitation spectra, the entrance slit was 1 nm, and the exit slit was 2.5 nm (pH 8.0) or 5 nm (pH 3.0).

### Crystallization

To prepare LSSmOrange microcrystals for SFX, a combination of seeding and batch methods was employed. First, crystal seeds were prepared by the sitting drop vapor diffusion technique using purified LSSmOrange at a concentration of about 20 mg/mL. The purified protein (1 μL or 2 μL) was mixed with an equal amount of precipitant (buffer A: 0.1 M Tris–HCl pH 8.0, 22–23% (w/v) PEG 3350, 0.9–1.1 M NaCl) in a 96-well Corning plate and was incubated at 20 °C. Large, plate-clustered crystals appeared within a few days and were subsequently used for the preparation of seed crystals (Figure S2A). The crystals were harvested together with the liquid in the droplet. Buffer A was added to rinse the well, and the mixture was combined with the harvested crystals. The mixture was then ground with a pestle for 1 min.

In order to obtain a sufficient quantity of microcrystals for the TR-SFX experiments, crystallization by the batch method was performed in 0.5 mL tubes. The mixture containing seed crystals (0.5–1 μL) was transferred to 0.5 mL tubes and mixed with precipitant (buffer B: 0.1 M Tris–HCl pH 8.0, 28% (w/v) PEG 3350, 0.6 M NaCl) (12–40 μL). Thereafter, the purified protein (4–10 μL) was added to the mixture so that the protein-to-buffer B ratio was 1:3 or 1:4. Each tube was then incubated at 20 °C. Finally, needle-like crystals with a length of 10 to 30 μm were obtained (Figure S2B). In total, 8.5 mL of microcrystals was prepared with a density of ca. 3.0 × 10^8^ crystals/ml. From the entire volume of microcrystals, 3.5 mL was used for SFX experiments.

### Synchrotron Diffraction Data Acquisition (Cryo-X-ray Crystallography)

For the data collection at SPring-8, LSSmOrange crystals were prepared in the same way as that for the SFX experiments. The microcrystals were concentrated by centrifugation and the removal of the supernatant. Crystallization buffer containing 20% ethylene glycol was added to the residual crystals. Subsequently, the mixture was transferred to 1 mm loops and rapidly frozen by using liquid nitrogen. The synchrotron X-ray diffraction data were collected at BL41XU^28^ using a 10 μm × 9 μm X-ray beam with a wavelength of 1 Å. A data set was collected with a total oscillation range of 5° and 0.25° oscillations per frame. Each frame was exposed to approximately 1.14 × 10^11^ photons. The resulting diffraction patterns from 300 to 400 crystals were processed using the KAMO^29^ suite, yielding a data set at a resolution of 1.91 Å. RADDOSE^30^ was used for the estimation of the dose rate of the data collected at SPring-8.

Diffraction images of crystals obtained from crystallization screening were checked at BL32XU^31^ and BL41XU.

### XFEL Diffraction Data Acquisition (RT SFX and Pump–Probe TR-SFX)

XFEL experiments were performed at BL3 (SACLA).^32,33^ The XFEL was operated with a repetition rate of 30 Hz, a photon energy of 10 keV, a pulse duration of <10 fs, and a focal spot of 1.5 μm at the full width at half-maximum (FWHM). A MPCCD-phase III detector^34^ was used at a sample-to-detector distance of 50 mm. A femtosecond pump pulse with a pulse duration of about 70 fs and a wavelength of 401 nm was supplied by the SACLA synchronized femtosecond laser system. The 50 μJ Gaussian beam pulses were focused onto an 80.7 μm × 88.9 μm (FWHM) exposure area at the intersecting point of the sample and the XFEL. In the pump–probe TR-SFX experiment, we collected the time-resolved data by setting the repetition rate of the pump laser to 15 Hz with alternating light and dark patterns. The pump laser’s temporal jitter monitored by the SACLA timing tool^35^ was approximately 50 fs (FWHM) with the recent synchronization upgrades.^36^ This temporal jitter was significantly smaller than the measurement time resolution (∼50 ps). Therefore, postprocessing sorting of the TR-SFX data was not required. The complete-dark data were collected without the pump laser illumination.

Prior to sample injection, 150 μL of LSSmOrange crystals in buffer B was ten times concentrated by centrifugation. This was followed by the addition of 250 μL of preprepared 16% hydroxyethyl cellulose.^13^ The resulting mixture was loaded into a high-viscosity cartridge-type injector^14^ that had a nozzle with an inner diameter of 75 μm. The mixture was ejected at a flow rate of 0.24 μL/min (without the pump laser) or 2.5 μL/min (with the pump laser). Diffraction images were filtered to extract “hit” images using Cheetah-pipeline software at SACLA.^37^ The detector geometry was refined by geoptimiser in CrystFEL.^38^ The recorded data were indexed by indexamajig of CrystFEL. Indexing was performed using XGANDALF, with peaks identified via PeakFinder8 using a threshold of 400, an integration radius of (3, 4, 7), and a minimum signal-to-noise ratio (SNR) of 3.5; all other parameters were retained at their default values. Then, the data were merged by the program process_*hkl* in CrystFEL with 3 iterations, no “push res” option by the Monte Carlo method.

Three XFEL data sets were acquired during the complete dark and alternating dark–light runs, which we call complete-dark, interleaved-dark, and light-250 ps, respectively. While the two dark data sets were cut at a high resolution of 1.7 Å, data up to 1.85 Å was included for the 250 ps data set. Data truncation was carried out using truncate.^39^ R-free flags were added with phenix.reflection_file_editor.^40^ Given the similarity between the complete-dark data and interleaved-dark data, the two data sets were merged into an all-dark data set which was also cut at resolution of 1.7 Å with truncate.^39^

### Optimization of Xtrapol8 Parameters, Calculation of Difference Fourier Maps, and Extrapolated Structure Factor Amplitudes

The computation of difference Fourier electron density maps and structure factor extrapolation was performed using Xtrapol8 v.1.2.0 - v1.2.3.^18,41,42^ Briefly, a Fourier difference map shows the difference between a reference and a triggered data set. Since the phases from the reference model are used to calculate the map, no assumption about the triggered state structure needs to be made. Structure factor amplitude extrapolation is a mathematical approach to estimate the structure factor amplitudes as if the crystals were fully occupied by the triggered state. Then, the extrapolated structure factor amplitudes can be used to refine the triggered state structure using conventional structure refinement tools.

To optimize the Xtrapol8 parameters, we first used the complete-dark data set as the reference data set (processed with CrystFEL initially with the “push-res = 1.25” option). We used the associated refined structure as the reference model and the 250 ps data set as the triggered data set. High isomorphism between the two data sets was observed up to a resolution of 2 Å after scaling the two data sets with isotropic *B*-factor scaling (overall *R*_iso_ = 0.10). We assessed various Bayesian-weighting schemes and extrapolated types provided within Xtrapol8. For weighting, we selected to test no weighting, *q*-weighting,^43^ and *k*-weighting,^44^ with *k*_scale_ varying between 0.05, 0.5, and 1.0, resulting in five weighting schemes. We also tested two extrapolation strategies, *F*_extr_ and *F*_extr_calc_, leading to a total of ten different extrapolation strategies. These were tested for a set of nine evenly spaced occupancies between 0.1 and 0.26 (or extrapolation factor α ranging from 10 to 3.8, see below). The optimal occupancy of each of them was estimated via automated methods within Xtrapol8. Given that only a single significant Fourier difference map peak exists on the carboxylate group of E215 (associated with decarboxylation), we realized that the difference-map-based methods were not sensitive enough to estimate the occupancy accurately. We therefore manually inspected all electron maps before and after refinement to come to a decision concerning the occupancy and optimal extrapolation parameters. We found that the occupancy of the triggered state would most likely lie between 0.12 and 0.16. While the difference Fourier map *F*_obs_^light‑250ps^–*F*_obs_^complete‑dark^ showed a stronger signal after *q*-weighting, the extrapolated electron density was easier to interpret when *k*-weighting with a *k*_scale_ factor of 0.5 was applied. Also, the maps were much clearer if *F*_extr_calc_ was used as compared to the *F*_extr_ scheme. We therefore identified the *F*_extr_calc_ scheme in combination with *k*-weighting with *k*_scale_ = 0.5 and occupancy of 0.12 (α = 8.33) as a valid extrapolation strategy for these data sets. The difference structure factor amplitudes and extrapolated structure factor amplitudes were calculated as

$$F_{diff}=\frac{k}{\left\langle k\right\rangle }\times \alpha \times \left(F_{obs}^{light}-F_{obs}^{dark}\right)$$ 1

$$F_{extr}=\frac{k}{\left\langle k\right\rangle }\times \alpha \times \left(F_{obs}^{light}-F_{obs}^{dark}\right)+F_{calc}^{dark}$$ 2

with

$$k={\left[1+\frac{\sigma_{\Delta F}^{2}}{{\langle \sigma }_{\Delta F}^{2}\rangle }+k_{scale}\frac{\Delta F^{2}}{\left\langle \Delta F^{2}\right\rangle }\right]}^{-1}$$

The respective Fourier difference electron density and extrapolated electron density were computed with the phase information on the reference state model.

It should be noted that different relations between the extrapolation factor α and the occupancy of the triggered state in the crystals have been reported. Here, we use the formalism by Genick^45^ which suggested a simple reciprocal relationship: occupancy=1α .

As a control, we also independently refined the light state structure using an alternative extrapolation strategy where we applied *q*-weighting, used the *F*_extr_ type of structure factor extrapolation and an occupancy of 0.16 (α = 6.25). The maps and refined structure point to the same structural changes as the structure described in the main text of this manuscript (Figure S7).

To verify that the interleaved dark images were not light-contaminated, we calculated a difference Fourier electron density map between the interleaved darks and the images collected during the full dark run (Figure S3). Since this electron density map does not contain any significant peaks, we also merged the complete-dark data with the interleaved-dark data. This data set we then call the all-dark data set. We then rerefined the dark state model in this stronger dark state data; used this newly refined structure and the all-dark data to redo the structure factor extrapolation. We used the Xtrapol8 parameters and strategy as optimized above for the complete-dark reference data. We again found a high isomorphism between the reference and triggered data sets and that an occupancy of 0.12 (α = 8.33) would be optimal in combination with the *F*_extr_calc_ strategy and *k*-weighting with *k*_scale_ = 0.5 weighting scheme. We note that better occupancy discrimination is achieved with the built-in difference-map method when the data were processed in CrystFEL without the “push-res” option.

### Structure Solution and Refinement

The ground-state structures obtained from the cryo-X-ray and SFX experiments were solved using the molecular replacement method in MOLREP.^46^ As a search model, 4Q7R (monomer A) from the PDB database was used after removal of the water molecules and the chromophore. The model was manually rebuilt and refined using Phenix^47^ and COOT.^48^ Restraints for the chromophore (three-letter code: OFM) were generated based on the chromophore in monomer A of 4Q7R with phenix.readyset. Restraints for the peptide links to the preceding and following amino acids were manually added based on the standard trans peptide bond. All refined structures were validated by MOLPROBITY.^49^ The refinement statistics are summarized in Table 1.

Structure refinement of the triggered state was carried out in a similar way using the extrapolated structure factor amplitudes. The dark state structure was applied as a reference model at the start, but reference restraints were omitted near the end to avoid model bias. Due to the decreased electron density map quality as compared to the dark state, several water molecules, residue side chains, and alternative conformations of some residues were deleted from the model. The relative occupancies of the two modeled E215 states (decarboxylated and altered conformation) were automatically refined in Phenix and converged to 54 and 46%, respectively.

We noted that the extrapolated data has a very low associated Wilson *B*-factor (9.2 Å^2^ as estimated by phenix.xtriage^40^ based on the given extrapolated structure factor amplitudes). This is caused by the low signal between 2.5 and 2.0 Å. Indeed, a Wilson *B*-factor of 23.9 Å^2^ is estimated for the data up to a high-resolution of 2.5 Å^2^. As a consequence, the atomic *B*-factors of the refined light state model are very low, as well. We therefore tried to apply a 2-fold *B*-factor blurring to the data using Servalcat,^50^ leading to a slightly increased Wilson *B*-factor of 11.2 Å^2^. Since this would only lead to a minor overall increase of the *B*-factors, we decided to not implement *B*-factor blurring. Also, we processed the raw data in CrystFEL with and without the “push-res” option, and noticed a minor improvement similar to *B*-factor blurring when the option was not used.

We also composed a model consisting of 88% of the dark state and 12% of the 250 ps state. In this model, residue 215 occupies the dark state for 88%, and the decarboxylated and alternate conformer states 6% each. This model was subsequently reciprocal space refined in the original 250 ps data, thereby refining the coordinates of the 250 ps state and the *B*-factors of both states. While the coordinates were not altered significantly as compared to the 250 ps model refined in extrapolated structure factor amplitudes (C_α_ superposition RMSD 0.15 Å), the average *B*-factor refined to 34.84 Å^2^, which is more in line with the Wilson *B*-factor. The refinement *R*_*work*_ and *R*_free_-values for this model are 16.28 and 20.24%, respectively. It is noteworthy that in this composite model, each of the two 250 ps conformations has an occupancy of 6%, which implies that they are not well resolved in the standard 2m*F*_obs_–D*F*_calc_ and m*F*_obs_–D*F*_calc_ electron density maps. This indicates the necessity of structure factor amplitude extrapolation to model the 250 ps state.

The structures and structure factor files have been deposited to the Protein Data Bank with accession codes 9LD5, 9LD8, and 9LD9.

### Femtosecond Transient Absorption Measurements

Experiments were performed using an amplified femtosecond double OPA laser system that provides three timed and spatially overlapping fs pulses at the sample position: an intense pulse (400 nm) was used for photoconversion, a second intense pulse was used to excite the sample (520 nm), and a weaker pulse was used to probe changes in absorption. The latter pulse was obtained via white light generation by focusing an 800 nm beam on a 3 mm sapphire plate to obtain a spectral quasi-continuum light in the visible region. The probe beam was sent to the polychromator to select the detection wavelength (560 nm) and was dispersed onto the PMT. This way, a sensitivity of less than 1 mOD was achieved. The pulse duration was 100 fs (FWHM) at the sample position, as determined by cross correlation. The measurements were performed under magic angle conditions (54.7° relative orientation between the two pump beams and probe light polarization planes).

Single-trace analysis was carried out using Origin’s nonlinear least-squares fitter (Originlab, Northampton, MA). A test exponential function convoluted with a Gaussian of 100 fs (FWHM, representative of the instrument temporal response function) was optimized to fit the experimental data sets. The quality of the fits was judged by the reduced χ^2^ values (within a given data set) as well as by visual inspection of the residuals.

### Computational Details

The constructed model used the RT SFX structure as the starting crystal structure, which included the chromophore, the E215 side chain, a number of neighboring amino acid side chains (K70, Q42, I197, L199, V73), and two crystal water molecules (HOH2, HOH4). The model was optimized by DFT using B3LYP/6-31g,^51–55^ Grimme’s D3 dispersion correction with the BJ damping function^56,57^ and geometrical constraints on the α-carbon of all amino acid side chains.^58^ On the optimized geometry of the model, TD-DFT calculations of excited states were performed with CAM-B3LYP­(D3BJ)/cc-pVDZ.^59,60^ In order to gain a clearer picture of the orbital transitions, natural transition orbitals were generated for the identified important excitations. All calculations were performed using Gaussian 16.^61^

## Supplementary Material



## Data Availability

The atomic models and structure factors generated in this study have been deposited in the Protein Data Bank (PDB) under accession codes 9LD5 (cryo-temperature data collected at SPring-8 BL41XU), 9LD8 (room-temperature data without pump laser), and 9LD9 (room-temperature data with 250 ps delayed pump laser). Additional PDB entries referenced in this work are publicly accessible through the PDB repository. Raw diffraction images supporting these findings have been archived in the Coherent X-ray Imaging Data Bank (CXIDB) under entry ID 235 (10.11577/2545657).

## Notes

Derivation of the transient absorption fitting function and additional details on the quantum-chemical calculations (PDF)

## References

[1] Kogure T., Karasawa S., Araki T., Saito K., Kinjo M., Miyawaki A. (2006). A fluorescent variant
of a protein from the stony coral
Montipora facilitates dual-color single-laser fluorescence cross-correlation
spectroscopy. Nat. Biotechnol..

[2] Piatkevich K. D., Hulit J., Subach O. M. (2010). Monomeric red fluorescent
proteins with a large Stokes shift. Proc. Natl.
Acad. Sci..

[3] Shcherbakova D. M., Hink M. A., Joosen L., Gadella T. W. J., Verkhusha V. V. (2012). An Orange
Fluorescent Protein with a Large Stokes Shift for Single-Excitation
Multicolor FCCS and FRET Imaging. J. Am. Chem.
Soc..

[4] Fron E., De Keersmaecker H., Rocha S. (2015). Mechanism Behind the
Apparent Large Stokes Shift in LSSmOrange Investigated by Time-Resolved
Spectroscopy. J. Phys. Chem. B.

[5] De
Keersmaecker H., Fron E., Rocha S. (2016). Photoconvertible
Behavior of LSSmOrange Applicable for Single Emission Band Optical
Highlighting. Biophys. J..

[6] Pletnev S., Shcherbakova D. M., Subach O. M., Pletneva N. V., Malashkevich V. N., Almo S. C., Dauter Z., Verkhusha V. V. (2014). Orange
Fluorescent Proteins: Structural Studies of LSSmOrange, PSmOrange
and PSmOrange2. PLoS One.

[7] Boutet S., Lomb L., Williams G. J. (2012). High-Resolution Protein
Structure Determination by Serial Femtosecond Crystallography. Science.

[8] Emma P., Akre R., Arthur J. (2010). First lasing and operation
of an ångstrom-wavelength free-electron laser. Nat. Photonics.

[9] Ishikawa T., Aoyagi H., Asaka T. (2012). A compact
X-ray free-electron
laser emitting in the sub-ångström region. Nat. Photonics.

[10] Barends T. R. M., Stauch B., Cherezov V., Schlichting I. (2022). Serial femtosecond
crystallography. Nat. Rev. Methods Prim.

[11] Brändén G., Neutze R. (2021). Advances and
challenges in time-resolved macromolecular
crystallography. Science.

[12] Owen R. L., Rudiño-Piñera E., Garman E. F. (2006). Experimental determination
of the radiation dose limit for cryocooled protein crystals. Proc. Natl. Acad. Sci..

[13] Sugahara M., Nakane T., Masuda T. (2017). Hydroxyethyl cellulose
matrix applied to serial crystallography. Sci.
Rep.

[14] Shimazu Y., Tono K., Tanaka T. (2019). High-viscosity sample-injection
device for serial femtosecond crystallography at atmospheric pressure. J. Appl. Crystallogr..

[15] Juers D. H., Matthews B. W. (2004). The role of solvent transport in
cryo-annealing of
macromolecular crystals. Acta Crystallogr. Sect
D Biol. Crystallogr..

[16] Neutze R., Wouts R., van der Spoel D., Weckert E., Hajdu J. (2000). Potential
for biomolecular imaging with femtosecond X-ray pulses. Nature.

[17] Francis W. J. C., Grewal H., Wainwright A. A. C., Yang X., Olivucci M., Miller R. J. D. (2024). Resonant multiphoton processes and excitation limits
to structural dynamics. Struct. Dyn..

[18] De
Zitter E., Coquelle N., Oeser P., Barends T. R. M., Colletier J. P. (2022). Xtrapol8 enables automatic elucidation of low-occupancy
intermediate-states in crystallographic studies. Commun. Biol..

[19] Sorigué D., Légeret B., Cuiné S. (2017). An algal photoenzyme
converts fatty acids to hydrocarbons. Science.

[20] Sorigué D., Hadjidemetriou K., Blangy S., Gotthard G., Bonvalet A., Coquelle N., Samire P., Aleksandrov A., Antonucci L., Benachir A. (2021). Mechanism and dynamics
of fatty acid photodecarboxylase. Science..

[21] Li X., Page C. G., Zanetti-Polzi L. (2023). Mechanism and Dynamics
of Photodecarboxylation Catalyzed by Lactate Monooxygenase. J. Am. Chem. Soc..

[22] Zhang X., Sun Y., Wang L., Li X., Zhong D. (2025). Ultrafast Dynamics
of Photoinduced Electron Transfer and Decarboxylation in the Flavoenzyme
Lactate Monooxygenase. J. Phys. Chem. B.

[23] Bell A. F., Stoner-Ma D., Wachter R. M., Tonge P. J. (2003). Light-Driven Decarboxylation
of Wild-Type Green Fluorescent Protein. J. Am.
Chem. Soc..

[24] Langhojer F., Dimler F., Jung G., Brixner T. (2009). Ultrafast Photoconversion
of the Green Fluorescent Protein Studied by Accumulative Femtosecond
Spectroscopy. Biophys. J..

[25] Čivić J., Mizuno H., Harvey J. N. (2025). Photoinduced
decarboxylation in fluorescent
proteins: charge-transfer states and structure–function relationship. Phys. Chem. Chem. Phys..

[26] Grigorenko B. L., Nemukhin A. V., Morozov D. I., Polyakov I. V., Bravaya K. B., Krylov A. I. (2012). Toward Molecular-Level Characterization of Photoinduced
Decarboxylation of the Green Fluorescent Protein: Accessibility of
the Charge-Transfer States. J. Chem. Theory
Comput..

[27] Ding L., Chung L. W., Morokuma K. (2013). Reaction Mechanism
of Photoinduced
Decarboxylation of the Photoactivatable Green Fluorescent Protein:
An ONIOM­(QM:MM) Study. J. Phys. Chem. B.

[28] Hasegawa K., Shimizu N., Okumura H. (2013). SPring-8 BL41XU, a high-flux
macromolecular crystallography beamline. J.
Synchrotron Radiat..

[29] Yamashita K., Hirata K., Yamamoto M. (2018). KAMO: towards automated
data processing
for microcrystals. Acta Crystallogr. Sect D
Struct Biol..

[30] Bury C. S., Brooks-Bartlett J. C., Walsh S. P., Garman E. F. (2018). Estimate your dose:
RADDOSE-3D. Protein Sci..

[31] Hirata K., Kawano Y., Ueno G. (2013). Achievement of protein
micro-crystallography at SPring-8 beamline BL32XU. J. Phys. Conf Ser..

[32] Ishikawa A., Tanaka A., Ikeda K. S., Shudo A. (2012). Diffraction and tunneling
in systems with mixed phase space. Phys. Rev.
E:Stat., Nonlinear, Soft Matter Phys..

[33] Tono K., Togashi T., Inubushi Y. (2013). Beamline,
experimental
stations and photon beam diagnostics for the hard x-ray free electron
laser of SACLA. New J. Phys..

[34] Kameshima T., Ono S., Kudo T., Ozaki K., Kirihara Y., Kobayashi K., Inubushi Y., Yabashi M., Horigome T., Holland A. (2014). Development of an X-ray
pixel detector with multi-port charge-coupled
device for X-ray free-electron laser experiments. Rev. Sci. Instrum..

[35] Katayama T., Owada S., Togashi T., Ogawa K., Karvinen P., Vartiainen I., Eronen A., David C., Sato T., Nakajima K. (2016). A beam branching method for timing and spectral
characterization of hard X-ray free-electron lasers. Struct. Dyn..

[36] Togashi T., Owada S., Kubota Y. (2020). Femtosecond Optical
Laser System with Spatiotemporal Stabilization for Pump-Probe Experiments
at SACLA. Appl. Sci..

[37] Nakane T., Joti Y., Tono K. (2016). Data processing pipeline
for serial femtosecond crystallography at SACLA. J. Appl. Crystallogr..

[38] White T. A., Kirian R. A., Martin A. V. (2012). CrystFEL:
a software
suite for snapshot serial crystallography. J.
Appl. Crystallogr..

[39] Evans P. R., Murshudov G. N. (2013). How good are my data and what is
the resolution?. Acta Crystallogr. Sect D Biol.
Crystallogr..

[40] Liebschner D., Afonine P. V., Baker M. L. (2019). Macromolecular structure
determination using X-rays, neutrons and electrons: recent developments
in Phenix. Acta Crystallogr. Sect D Struct Biol..

[41] Liebschner D., Afonine P. V., Moriarty N. W. (2017). Polder maps: improving
OMIT maps by excluding bulk solvent. Acta Crystallogr.
Sect D Struct Biol..

[42] Winn M. D., Ballard C. C., Cowtan K. D. (2011). Overview
of the CCP
4 suite and current developments. Acta Crystallogr.
Sect D Biol. Crystallogr..

[43] Ursby T., Bourgeois D. (1997). Improved Estimation of Structure-Factor
Difference
Amplitudesfrom Poorly Accurate Data. Acta Crystallogr.
Sect A Found Crystallogr..

[44] Ren Z., Perman B., Šrajer V. (2001). A Molecular Movie at
1.8 Å Resolution Displays the Photocycle of Photoactive Yellow
Protein, a Eubacterial Blue-Light Receptor, from Nanoseconds to Seconds. Biochemistry.

[45] Genick U. K. (2007). Structure-factor
extrapolation using the scalar approximation: theory, applications
and limitations. Acta Crystallogr. Sect D Biol.
Crystallogr..

[46] Vagin A., Teplyakov A. (2010). Molecular replacement with MOLREP. Acta Crystallogr. Sect D Biol. Crystallogr..

[47] Adams P. D., Afonine P. V., Bunkóczi G. (2010). PHENIX: a comprehensive
Python-based system for macromolecular structure solution. Acta Crystallogr. Sect D Biol. Crystallogr..

[48] Emsley P., Cowtan K. (2004). Coot: model-building
tools for molecular graphics. Acta Crystallogr.
Sect D Biol. Crystallogr..

[49] Chen V. B., Arendall W. B., Headd J. J. (2010). MolProbity:
all-atom
structure validation for macromolecular crystallography. Acta Crystallogr. Sect D Biol. Crystallogr..

[50] Yamashita K., Wojdyr M., Long F., Nicholls R. A., Murshudov G. N. (2023). GEMMI and
Servalcat restrain REFMAC 5. Acta Crystallogr.
Sect D Struct Biol..

[51] Becke A. D. (1993). Density-functional
thermochemistry. III. The role of exact exchange. J. Chem. Phys..

[52] Lee C., Yang W., Parr R. G. (1988). Development of the Colle-Salvetti
correlation-energy formula into a functional of the electron density. Phys. Rev. B.

[53] Vosko S. H., Wilk L., Nusair M. (1980). Accurate spin-dependent electron
liquid correlation energies for local spin density calculations: a
critical analysis. Can. J. Phys..

[54] Stephens P. J., Devlin F. J., Chabalowski C. F., Frisch M. J. (1994). Ab Initio Calculation
of Vibrational Absorption and Circular Dichroism Spectra Using Density
Functional Force Fields. J. Phys. Chem..

[55] Krishnan R., Binkley J. S., Seeger R., Pople J. A. (1980). Self-consistent
molecular orbital methods. XX. A basis set for correlated wave functions. J. Chem. Phys..

[56] Grimme S., Antony J., Ehrlich S., Krieg H. (2010). A consistent and accurate
ab initio parametrization of density functional dispersion correction
(DFT-D) for the 94 elements H-Pu. J. Chem. Phys..

[57] Grimme S., Ehrlich S., Goerigk L. (2011). Effect of the damping function in
dispersion corrected density functional theory. J. Comput. Chem..

[58] Siegbahn P. E. M., Himo F. (2011). The quantum chemical cluster approach for modeling
enzyme reactions. WIREs Comput. Mol. Sci..

[59] Yanai T., Tew D. P., Handy N. C. (2004). A new hybrid
exchange–correlation
functional using the Coulomb-attenuating method (CAM-B3LYP). Chem. Phys. Lett..

[60] Dunning T. H. (1989). Gaussian
basis sets for use in correlated molecular calculations. I. The atoms
boron through neon and hydrogen. J. Chem. Phys..

[61] Frisch, M. J. ; Trucks, G. W. ; Schlegel, H. B. ; Gaussian16. Rev. C.01: Wallingford, CT, 2016.

