# Supplementary material for: Decarboxylation via a Higher Electronic Excited State Drives LSSmOrange Photoconversion
Source: ACS Phys Chem Au. 2026 Apr 12;6(3):534–51. doi: 10.1021/acsphyschemau.6c00009 (PMC13220194; doi:10.1021/acsphyschemau.6c00009)
Supplement: Supplementary file 1 [file pg6c00009_si_001.pdf]

Supporting Information

Decarboxylation via a higher electronic excited state drives LSSmOrange photoconversion

Hyang Sook Seol[‡a], Fangjia Luo[‡b], Elke De Zitter[c], Nipawan Nuemket[b,d], Eduard Fron[e], Neil R. McFarlane[f], Leonie De Vrieze[a], Janko Civic[f], Michiel Postelmans[a], Savannah Van Bel[a], Shigeki Owada[b,d], Kensuke Tono[b,d], Tomoyuki Tanaka[d], Toshi Arima[d], Hiroki Noguchi[g], Thi Yen Hang Bui[h], Rie Tanaka[d,i], Kazuya Hasegawa[b,d], Kunio Hirata[d], Dohyun Im[i], Tsuyoshi Araya[i], Tetsunari Kimura[j], Luc Van Meervelt[h], Martin Weik[c], Jeremy N. Harvey[f], Jacques-Philippe Colletier[c], So Iwata*[d,i], Eriko Nango*[i,k], Hideaki Mizuno*[a]

---

a. Laboratory of Biomolecular Network Dynamics, Biochemistry, Molecular and Structural Biology Section, Department of Chemistry, KU Leuven, Celestijnenlaan 200G bus 2403, 3001 Leuven, Belgium

b. Japan Synchrotron Radiation Research Institute, 1-1-1 Kouto, Sayo, Hyogo 679-5198, Japan

c. Univ. Grenoble Alpes, CEA, CNRS, Institut de Biologie Structurale 38000 Grenoble, France

d. SPring-8 Center, RIKEN, 1-1-1 Kouto, Sayo-cho, Sayo-gun, Hyogo, 679-5148, Japan

e. Chem&Tech-Molecular Imaging and Photonics, Department of Chemistry, KU Leuven, Celestijnenlaan 200F bus 2404, 3001 Leuven, Belgium

f. Quantum Chemistry and Physical Chemistry Section, Department of Chemistry, KU Leuven, Celestijnenlaan 200F bus 2404, 3001 Leuven, Belgium

g. Laboratory of Biomolecular modelling and design, Biochemistry, Molecular and Structural Biology Section, Department of Chemistry, KU Leuven, Celestijnenlaan 200G bus 2403, 3001 Leuven, Belgium

h. Laboratory of Biomolecular Architecture, Biochemistry, Molecular and Structural Biology Section, Department of Chemistry, KU Leuven, Celestijnenlaan 200G bus 2403, 3001 Leuven, Belgium

i. Department of Cell Biology, Graduate School of Medicine, Kyoto University, Yoshidakonoe-cho, Sakyo-ku, Kyoto 606-8501, Japan

j. Department of Chemistry, Graduate School of Science, Kobe University 1-1 Rokkodai, Nada, Kobe 657-8501, Japan

k. Institute of Multidisciplinary Research for Advanced Materials, Tohoku University, Aoba-ku, Sendai 980-8577, Japan

---

‡ These authors contribute equally to this work

*Correspondence: iwata.so.2z@kyoto-u.ac.jp; eriko.nango.c4@tohoku.ac.jp; hideaki.mizuno@kuleuven.be

Table of contents

Text S1: Evaluation of the photoconversion process by transient absorption

Text S2. Additional details quantum-chemical calculations

Table S1. The five lowest-lying excited states for the largest cluster model calculated at the CAM-B3LYP(D3BJ)/cc-pVDZ level of theory

Fig. S1. Unnormalized fluorescence spectra of LSSmOrange at pH 8.0 under cryogenic conditions

Fig. S2. Crystals used for the preparation of seed crystals and microcrystals employed for structure determination by cryo-X-ray crystallography, RT SFX and pump-probe TR-SFX

Fig. S3. $F_{obs,}^{interleaved\text{-}dark}$-$F_{obs,}^{complete\text{-}dark}$ Fourier difference map showing no differences between the interleaved and complete-dark images

Fig. S4. Re-refinement of the dark state

Fig. S5. Refinement in the presence of CO_2

Fig. S6. Backbone shift in the 250 ps structure

Fig. S7. q-weighting

Fig. S8. Enhanced LSSmOrange photoconversion via multiphoton absorption process

Fig. S9. Representation of the three models used in the quantum chemical calculations

Text S1: Evaluation of the photoconversion process by transient absorption

From Fick's first diffusion law, the flux across the interface of the observation volume (J) is described as:

$$J = -D \cdot \frac{\partial[C]}{\partial r} \qquad (1)$$

The concentration gradient is described as follows:

$$\frac{\partial[C]}{\partial r} = \frac{[C_{in}] - [C_{out}]}{r_{in} - r_{out}} \qquad (2)$$

where $[C_{in}]$ and $[C_{out}]$ are the concentrations of the fluorescent protein inside and outside the observation area and r_{in} and r_{out} are the radii from the center of the observation volume to the inside or outside of the surface of the observation volume.

Change in $[C_{in}]$ by diffusion is described with:

$$\left(\frac{d[C_{in}]}{dt}\right)_{diff} = \frac{J \cdot A}{V} \qquad (3)$$

where A is the surface area of the observation volume and V the volume of observation volume.

Putting (1) in (3)

$$\left(\frac{d[C_{in}]}{dt}\right)_{diff} = -\frac{A}{V} \cdot D \cdot \frac{\partial[C]}{\partial r} \qquad (4)$$

Putting (2) in (4)

$$\left(\frac{d[C_{in}]}{dt}\right)_{diff} = -\frac{A}{V} \cdot D \cdot \frac{[C_{in}] - [C_{out}]}{r_{in} - r_{out}} \qquad (5)$$

Use (5) to describe the change in concentration of photoconverted LSSmOrange ($[PC_{in}]$):

$$\left(\frac{d[PC_{in}]}{dt}\right)_{diff} = -\frac{A}{V} \cdot D_{PC} \cdot \frac{[PC_{in}] - [PC_{out}]}{r_{in} - r_{out}} \qquad (6)$$

where D_{PC} is the diffusion coefficient of the photoconverted species. Since there are almost no photoconverted LSSmOrange molecules outside the observation area: $[PC_{out}] \approx 0$

$$\left(\frac{d[PC_{in}]}{dt}\right)_{diff} = -\frac{A}{V} \cdot D_{PC} \cdot \frac{1}{r_{in} - r_{out}} \cdot [PC_{in}] \qquad (7)$$

$$set\ k_{diff,PC} = \frac{A}{V} \cdot D_{PC} \cdot \frac{1}{r_{in} - r_{out}}$$

$$\left(\frac{d[PC_{in}]}{dt}\right)_{diff} = -k_{diff,PC} \cdot [PC_{in}] \qquad (8)$$

Use (5) to describe the change in concentration of unconverted LSSmOrange ($[N_{in}]$)

$$\left(\frac{d[N_{in}]}{dt}\right)_{diff} = -\frac{A}{V} \cdot D_N \cdot \frac{[N_{in}] - [N_{out}]}{r_{in} - r_{out}} \qquad (9)$$

Since the observation volume is significantly small compared to the entire volume of the sample solution, the change in the total concentration of the unconverted species is negligible ($[N_{out}] \approx [N_0]$)

$$\left(\frac{d[N_{in}]}{dt}\right)_{diff} = -\frac{A}{V} \cdot D_N \cdot \frac{1}{r_{in} - r_{out}} \cdot [N_{in}] + \frac{A}{V} \cdot D_N \cdot \frac{[N_0]}{r_{in} - r_{out}} \qquad (10)$$

$$set\ k_{diff,N} = \frac{A}{V} \cdot D_N \cdot \frac{1}{r_{in} - r_{out}}$$

$$\left(\frac{d[N_{in}]}{dt}\right)_{diff} = -k_{diff,N}[N_{in}] + k_{diff,N} \cdot [N_0] \qquad (11)$$

where $k_{diff,N} \cdot [N_0]$ indicates influx of unconverted species into the illumination volume.

Changes in concentration by photoconversion are described as follows:

$$\left(\frac{d[PC_{in}]}{dt}\right)_{pc} = k_{pc} \cdot [{N_{in}}^*] \qquad (12)$$

$$\left(\frac{d[N_{in}]}{dt}\right)_{pc} = -k_{pc} \cdot [{N_{in}}^*] \qquad (13)$$

where $[{N_{in}}^*]$ is the concentration of unconverted species in the excited state in the observation volume. $[{N_{in}}^*]$ linearly correlates to the ground state concentration of the unconverted species:

$$[{N_{in}}^*] = [N_{in}] \cdot p \qquad (14)$$

where p is the probability of the transition of the unconverted species to the excited state. Changes in concentration by photoconversion are described as follows:

$$\left(\frac{d[PC_{in}]}{dt}\right)_{pc} = k_{pc} \cdot [N_{in}] \cdot p \qquad (15)$$

$$\left(\frac{d[N_{in}]}{dt}\right)_{pc} = -k_{pc} \cdot [N_{in}] \cdot p \qquad (16)$$

Total change in $[PC_{in}]$ and $[N_{in}]$ are as follows:

$$\frac{d[PC_{in}]}{dt} = \left(\frac{d[PC_{in}]}{dt}\right)_{pc} + \left(\frac{d[PC_{in}]}{dt}\right)_{diff} = k_{pc} \cdot [N_{in}] \cdot p - k_{diff,PC} \cdot [PC_{in}] \qquad (17)$$

$$\frac{d[N_{in}]}{dt} = \left(\frac{d[N_{in}]}{dt}\right)_{pc} + \left(\frac{d[N_{in}]}{dt}\right)_{diff} = -k_{pc} \cdot [N_{in}] \cdot p - k_{diff,N}[N_{in}] + k_{diff,N} \cdot [N_0] \qquad (18)$$

At steady state:

From (18):

$$\frac{d[N_{in,ss}]}{dt} = -k_{pc} \cdot [N_{in,ss}] \cdot p - k_{diff,N}[N_{in,ss}] + k_{diff,N} \cdot [N_0] = 0$$

$$(k_{pc} \cdot p + k_{diff,N}) \cdot [N_{in,ss}] = k_{diff,N} \cdot [N_0]$$

$$[N_{in,ss}] = \frac{k_{diff,N} \cdot [N_0]}{k_{pc} \cdot p + k_{diff,N}} \qquad (19)$$

From (17):

$$\frac{d[PC_{in,ss}]}{dt} = k_{pc} \cdot [N_{in,ss}] \cdot p - k_{diff,PC} \cdot [PC_{in,SS}] = 0$$

$$k_{pc} \cdot [N_{in,ss}] \cdot p = k_{diff,PC} \cdot [PC_{in,SS}]$$

$$[PC_{in,SS}] = \frac{k_{pc} \cdot p}{k_{diff,PC}} \cdot [N_{in,ss}] \qquad (20)$$

Put (19) into (20)

$$[PC_{in,SS}] = \frac{k_{pc} \cdot p}{k_{diff,PC}} \cdot \frac{k_{diff,N} \cdot [N_0]}{k_{pc} \cdot p + k_{diff,N}}$$

$$[PC_{in,SS}] = \frac{k_{diff,N} \cdot [N_0]}{k_{diff,PC}} \cdot \frac{k_{pc} \cdot p}{k_{pc} \cdot p + k_{diff,N}}$$

$$[PC_{in,SS}] = \frac{k_{diff,N} \cdot [N_0]}{k_{diff,PC}} \cdot \left(1 - \frac{k_{diff,N}}{k_{pc} \cdot p + k_{diff,N}}\right)$$

$$[PC_{in,SS}] = \frac{k_{diff,N} \cdot [N_0]}{k_{diff,PC}} \cdot \left(1 - \frac{1}{\frac{k_{pc}}{k_{diff,N}} \cdot p + 1}\right) \qquad (21)$$

$|\Delta OD| - |\Delta OD_{off}|$ linearly correlates to $[PC_{in}]$:

$$[PC_{in}] = \frac{1}{\varepsilon l} \cdot (|\Delta OD| - |\Delta OD_{off}|) \qquad (22)$$

where $|\Delta OD|$ is the absolute value of the transient absorption, $|\Delta OD_{off}|$ is the background obtained without illumination of the photoconversion laser, ε is the extinction coefficient and l the path length. At steady state:

$$[PC_{in,SS}] = \frac{1}{\varepsilon l} \cdot (|\Delta OD_{ss}| - |\Delta OD_{off}|) \qquad (23)$$

Compare (23) and (21)

$$\left(|\Delta OD_{ss}| - |\Delta OD_{off}|\right) = \varepsilon l \cdot \frac{k_{diff,N} \cdot [N_0]}{k_{diff,PC}} \cdot \left(1 - \frac{1}{\frac{k_{pc}}{k_{diff,N}} \cdot p + 1}\right) \qquad (24)$$

The probability of the transition to the excited state correlates to the photon density in the case of a single-photon process, square of the photon density in the case of a two-photon process, and cubic of the photon density in the case of a three-photon process.

$$p = \sigma[\gamma]^n \qquad (25)$$

where $[\gamma]$ indicates the photon density, and σ is the cross-section. Since $[\gamma]$ linearly correlates to the pulse energy of the laser (E)

$$[\gamma] = \frac{\lambda}{hc} \cdot \frac{1}{A_I} \cdot E \qquad (26)$$

where A_I is the area of the laser spot

$$[\gamma]^n = \left(\frac{\lambda}{hc} \cdot \frac{1}{A_I}\right)^n \cdot E^n \qquad (27)$$

Put (27) into (25)

$$p = \sigma[\gamma]^n = \sigma\left(\frac{\lambda}{hc} \cdot \frac{1}{A_I}\right)^n \cdot E^n \qquad (28)$$

$$set\ k'_{pc} = k_{pc} \cdot \sigma\left(\frac{\lambda}{hc} \cdot \frac{1}{A_I}\right)^n$$

$$p = \frac{k'_{pc}}{k_{pc}} \cdot E^n \qquad (29)$$

Put (29) into (24)

$$\left(|\Delta OD_{ss}| - |\Delta OD_{off}|\right) = \varepsilon l \cdot \frac{k_{diff,N} \cdot [N_0]}{k_{diff,PC}} \cdot \left(1 - \frac{1}{\frac{k'_{pc}}{k_{diff,N}} \cdot E^n + 1}\right) \qquad (30)$$

$$set\ A_1 = \varepsilon l \cdot \frac{k_{diff,N} \cdot [N_0]}{k_{diff,PC}}\ and\ A_2 = \frac{k'_{pc}}{k_{diff,N}}$$

$$\left(|\Delta OD_{ss}| - |\Delta OD_{off}|\right) = A_1\left(1 - \frac{1}{A_2 E^n + 1}\right)$$

where n indicates the nonlinear susceptibility. The $\left(|\Delta OD_{ss}| - |\Delta OD_{off}|\right)$ vs E curve can be fit to the function expressed in equation (30).

Text S2: Additional details quantum-chemical calculations

Calculations describing the electronic structure of the ground and excited states of the LSSmOrange protein were performed using "cluster" models of the protein, in which the residues forming the chromophore were included, together with some atoms from the protein environment, with the rest of the protein omitted. These calculations do not provide a quantitative description of the system, but provided that a large enough model is used, so that the key electrostatic interactions are present, do provide a qualitatively correct prediction of the nature of the excited states.

After selecting the atoms to include in the model, structural optimization at the DFT level using B3LYP/6-31g was performed as indicated in the main text, with some constraints on the position of side-chain atoms in order to reproduce the effects of the protein matrix, as illustrated in Figure S9. The excited-states were then calculated at the CAM-B3LYP(D3BJ)/cc-pVDZ level of theory.

As an initial validation, a very small model that included only the chromophore was constructed (Fig. S9a), where the first excited state involved a HOMO to LUMO transition with a predicted absorption wavelength of 375 nm and a large oscillator strength – in good agreement with experiment. Not including the E215 side chain implies that the charge transfer processes of the second excitation could not be captured. Subsequent exploratory calculations on an extended, albeit still small, model in which the E215 side chain and a nearby neutralizing K70 side chain were included (Figure S9b), led to confusing results as the relative energy of the carboxylate orbitals were poorly rendered due to an improper description of the surrounding environment, such that the lowest-energy excited state was no longer chromophore-centered. To improve carboxylate orbital rendering, a larger model including the E215 side chain, nearby crystal structure water molecules and a number of amino acid side chains surrounding E215 was constructed (FigS9c) and a better description of the environment effects was obtained, as described in the main text. The data for the five lowest-lying excited states of this model are shown in Table S1.

Table S1. The five lowest-lying excited states for the largest cluster model calculated at the CAM-B3LYP(D3BJ)/cc-pVDZ level of theory.

Excited State	Energy (eV)	Energy (nm)	Oscillator Strength (a.u.)	Character
**1**	**3.3230**	**373.11**	**0.5916**	**Chromophore → chromophore**
2	3.6873	336.25	0.0046	Environment → chromophore
3	4.1393	299.53	0.0006	Environment → chromophore
**4**	**4.3381**	**285.81**	**0.0064**	**E215 → chromophore**
5	4.4973	275.68	0.0514	Environment → chromophore

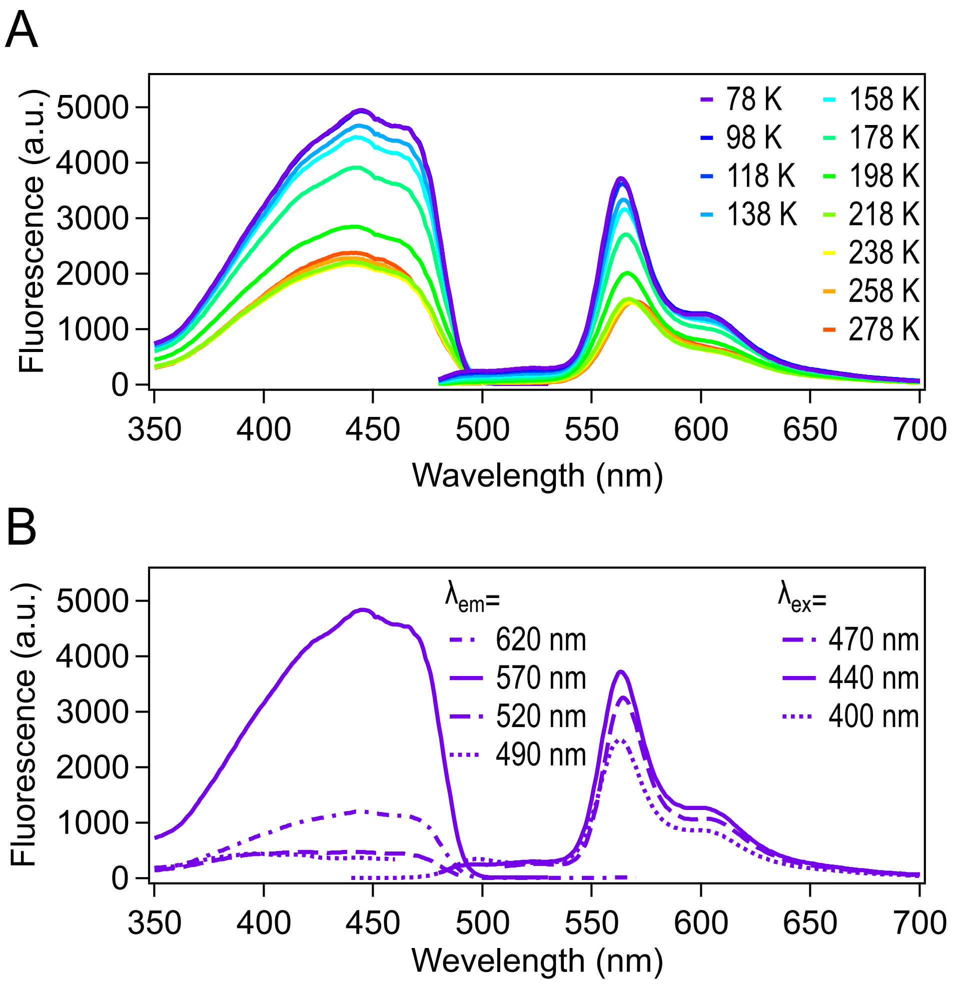

Fig. S1. Unnormalized fluorescence spectra of LSSmOrange at pH 8.0 under cryogenic conditions. A. Unnormalized excitation (left) and emission (right) spectra at various temperatures. The excitation spectra were recorded with an emission wavelength of 570 nm. The emission spectra were recorded using excitation at 440 nm. LSSmOrange in solution (64 µM) in 50 mM HEPES buffer (pH 8.0) was used for the measurement. **B.** Unnormalized fluorescence spectra at 78 K. Excitation spectra were recorded with an emission wavelength of 490 nm (dotted line), 520 nm (dashed line), 570 nm (solid line) or 620 nm (chained line). Emission spectra were recorded using excitation at 400 nm (dotted line), 440 nm (solid line) or 470 nm (dashed line).

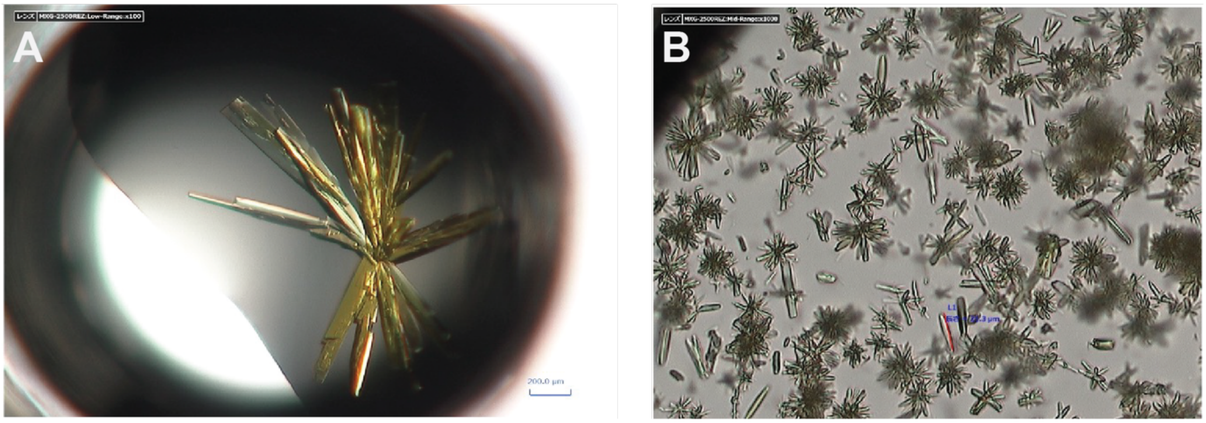

Fig. S2. Crystals used for the preparation of seed crystals and microcrystals employed for structure determination by cryo-X-ray crystallography, RT SFX and pump-probe TR-SFX. A. Large plate-clustered crystals were grown in 0.1 M Tris-HCl pH 8.0, 22-23% (w/v) PEG 3350, 0.9 - 1.1 M NaCl using the sitting drop vapor diffusion method and were ground to yield seed crystals. **B.** The seed crystals formed the starting point to grow microcrystals using the batch method (0.1 M Tris-HCl pH 8.0, 28% (w/v) PEG 3350, 0.6 M NaCl).

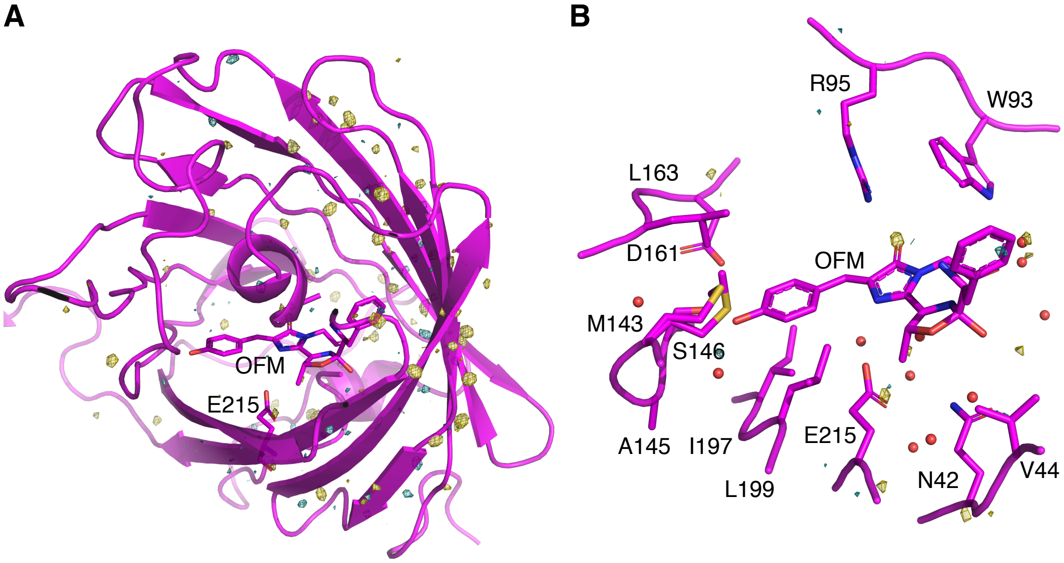

Fig. S3. $F_{\mathrm{obs},}^{\mathrm{interleaved\text{-}dark}}$-$F_{\mathrm{obs},}^{\mathrm{complete\text{-}dark}}$ Fourier difference map showing no differences between the interleaved and complete-dark images. The map is contoured at ± 4 r.m.s.d.; teal: positive electron density; yellow: negative electron density. The dark state structure is shown in magenta. **A.** Global view over the full protein. The long N-terminal was omitted for clarity. **B.** Zoom in on the chromophore pocket.

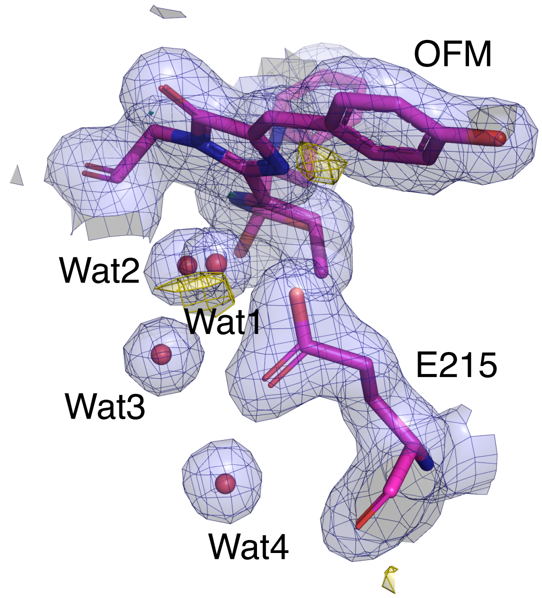

Fig. S4. Re-refinement of the dark state. The dark state was re-refined using all dark images. Magenta: dark state model, blue: 2mF_{obs}-DF_{calc} electron density contoured at 1 r.m.s.d.; teal and yellow: mF_{obs}-DF_{calc} electron density contoured at ± 3 r.m.s.d.

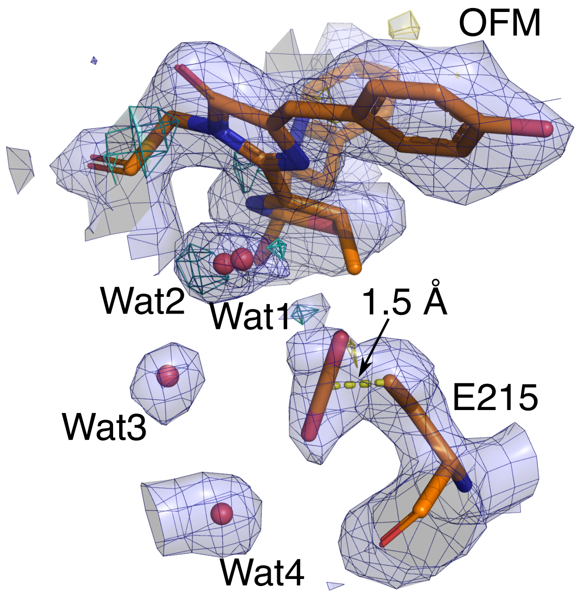

Fig. S5. Refinement in the presence of CO_2. The 250 ps state structure was refined with a CO_2 group present in the density next to E215 (blue: refined 2mF_{extr}-DF_{calc}, 1 r.m.s.d.; teal/yellow refined mF_{extr}-DF_{calc} contoured at ± 3 r.m.s.d.). Phenix.refine imposed a bond between the CO_2 group and E215 because of the short distance between the two carbon atoms. In addition, the linear geometry of the CO_2 group does not optimally fit the electron density. Imposing a longer distance between the CO_2 group and E215 does not lead to a geometry in which the CO_2 group adequately fits the electron density.

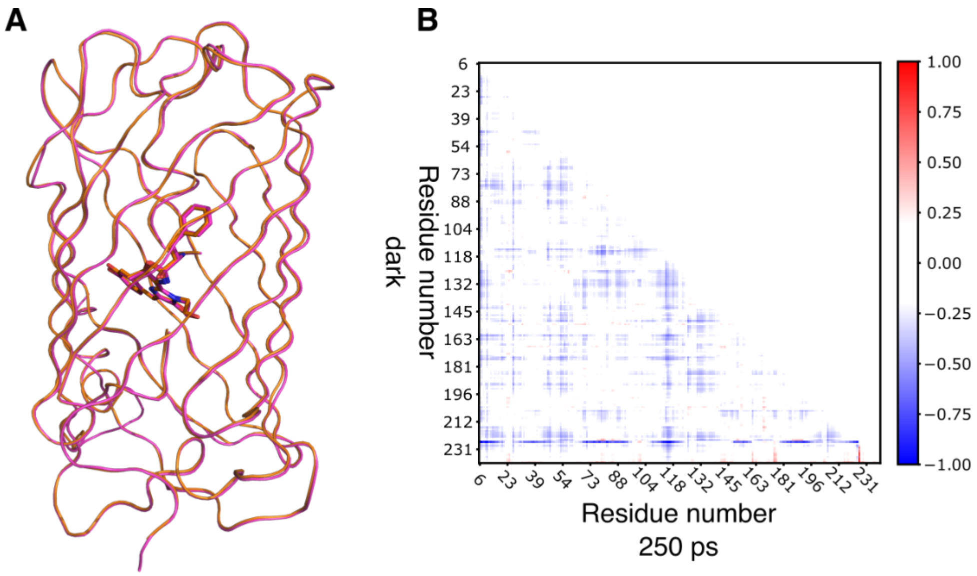

Fig. S6. Minimal structural changes between the dark and the 250 ps structure. A. Superposition of the 250 ps structure (orange) on the dark state structure (magenta) indicated that the 250 ps structure has a slight backbone shift as compared to the dark state structure. **B.** Difference distance matrix (ddm) using the dark state and 250 ps structure's Cα atoms. It shows that the 250 ps structure is overall more compact than the dark state structure, with a Cα superposition r.m.s.d. value of 0.2 Å (for comparison, the r.m.s.d. value between the structures refined against the "*interleaved-dark" and "complete-dark" datasets* was only 0.02 Å).

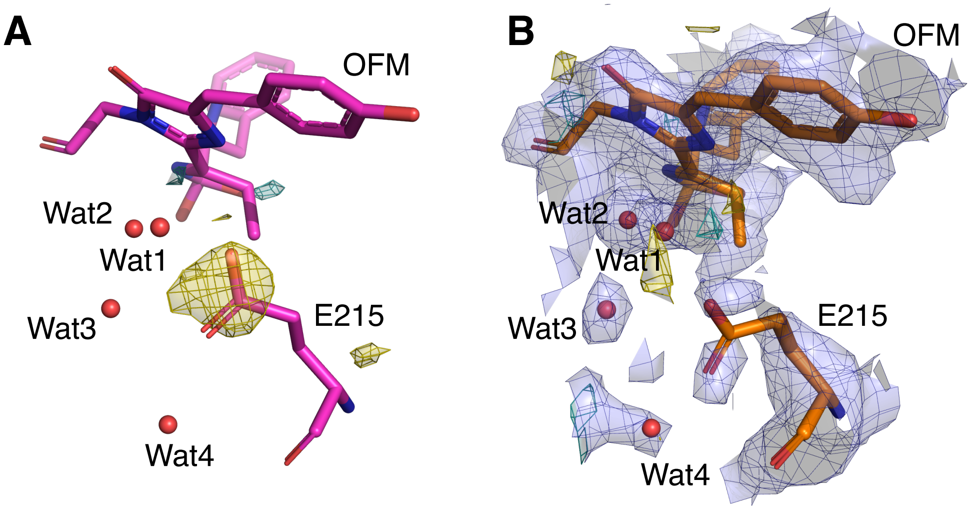

Fig. S7. q-weighting. A. Fourier difference ($F_{obs,}{}^{light\text{-}250ps}$ - $F_{obs,}{}^{complete\text{-}dark}$) calculated with *q-weighting* and an occupancy of 16% shows the same features as the Fourier difference map calculated with *k-weighting* with occupancy of 12% (contour level ± 4 r.m.s.d.; teal: positive electron density; yellow: negative electron density; magenta: dark state model). **B.** The structure refined in the extrapolated structure factor amplitudes calculated with q-weighting and an occupancy of 16% shows a partially decarboxylated (49%) and partial alternative conformation (51%) for the E215 side chain (blue: refined 2mF_{extr}-DF_{calc}, 1 r.m.s.d.; teal/yellow refined mF_{extr}-DF_{calc} contoured at ± 3 r.m.s.d.).

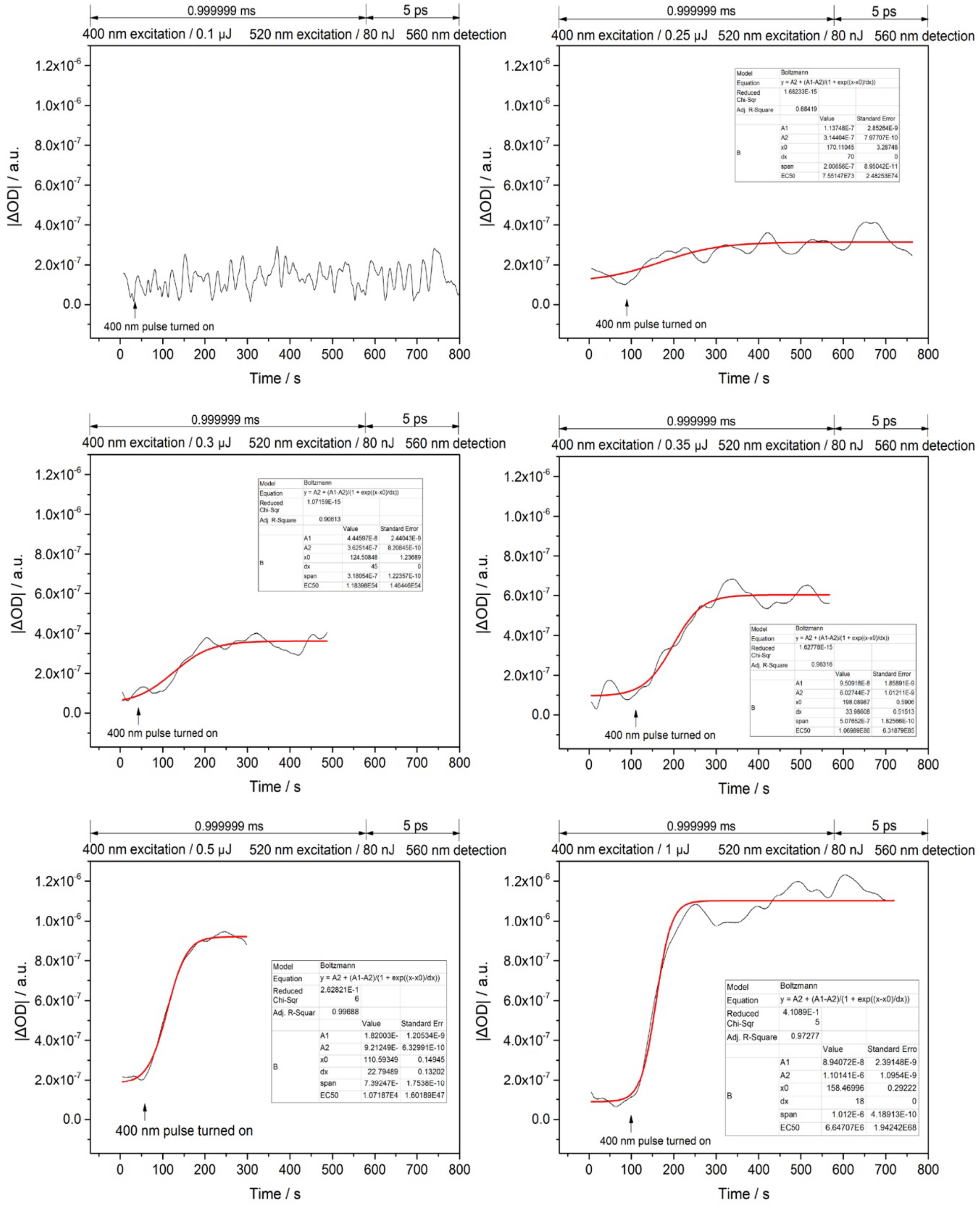

Fig. S8. Enhanced LSSmOrange photoconversion via multiphoton absorption process. Accumulation of photoconverted LSSmOrange by repetitive illumination with a 100-fs pulse laser at 400 nm. The repetition rate of the photoconversion laser was 1 kHz. The amount of photoconverted LSSmOrange was estimated by exciting the photoconverted species with a 520 nm pump laser and by detecting the combination of the ground state absorption and stimulated emission signals at 560 nm ($|\Delta OD|$). The measurement was repeated with various pulse energies ranging from 0.1 μJ to 1.0 μJ.

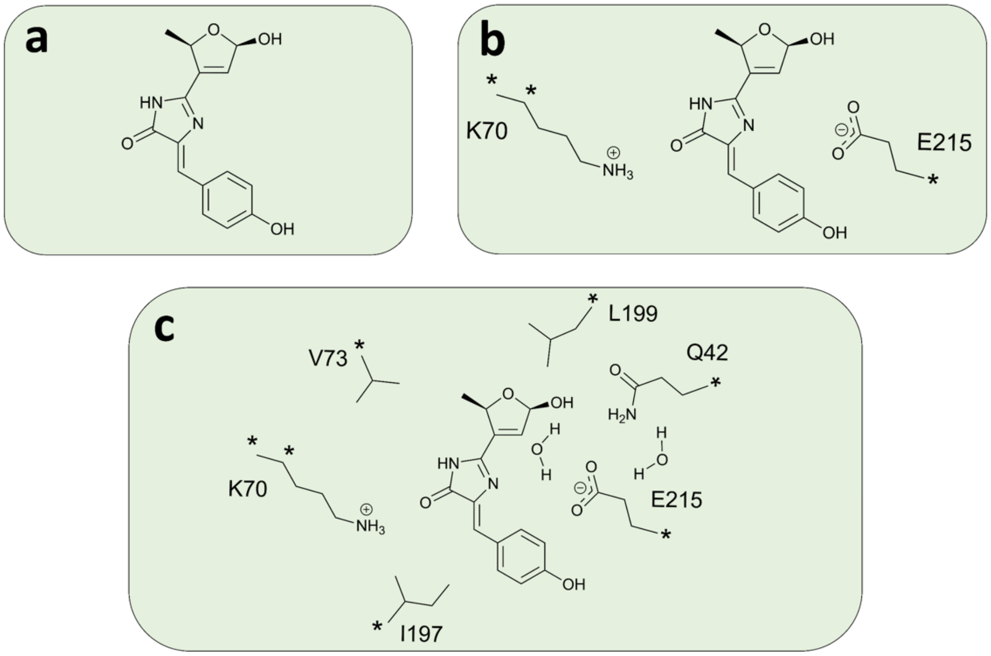

Fig. S9. Representation of the three models used in the quantum chemical calculations. Amino acids are all labelled and the constrained atoms are indicated by stars.
